# A synergistic nanozyme platform breaking the neuroinflammatory-oxidative stress cycle for extended pain relief

**DOI:** 10.1016/j.ijpx.2026.100561

**Published:** 2026-05-05

**Authors:** Chengfeng Zhang, Zihan Xue, Jingyi Wang, Jianing Li, Wanlong Qian, Xueting Wang, Yong-Jing Gao, Zhongping Chen, Faming Wang, Yan Zhang

**Affiliations:** Institute of Special Environmental Medicine, School of Public Health, Nantong University, Nantong 226019, China

**Keywords:** Chronic pain, Reactive nitrogen and oxygen species, Liposome, Nanozyme, Raddeanin a

## Abstract

Chronic pain represents a major public health burden that significantly compromises patient quality of life. Its persistence is driven by a vicious cycle of neuroinflammation and oxidative stress within the nervous system. Effective clinical management critically requires targeted therapeutics with long-lasting efficacy. Herein, we report a novel liposome-based co-delivery system (SeCQDs@Liposome-RA, SLR) encapsulating selenium-doped carbon quantum dots (SeCQDs) nanozymes and the potent anti-inflammatory agent Raddeanin A (RA). This platform was rationally engineered for synergistic antioxidant and anti-inflammatory actions. *In vitro*, SLR nanoparticles efficiently neutralized reactive nitrogen and oxygen species and significantly suppressed pro-inflammatory cytokines in activated microglia. Crucially, leveraging the liposome's sustained-release capabilities, a single dose of SLR provided profound and durable analgesia in a Complete Freund's Adjuvant-induced inflammatory pain mouse model, lasting up to 72 h. Mechanistically, SLR disrupted the neuroinflammatory cycle by inhibiting the mitogen-activated protein kinase/nuclear factor kappa B signaling pathways, thereby suppressing microglial overactivation and mitigating oxidative stress within the spinal cord and brain. Furthermore, SLR demonstrated broad-spectrum efficacy in chemotherapy- and spared nerve injury-induced neuropathic pain models. Given its exceptional biosafety and sustained-release characteristics, SLR represents a versatile and clinically promising nanoplatform for the long-term management of chronic pain syndromes.

## Graphical abstract

**Figure:**
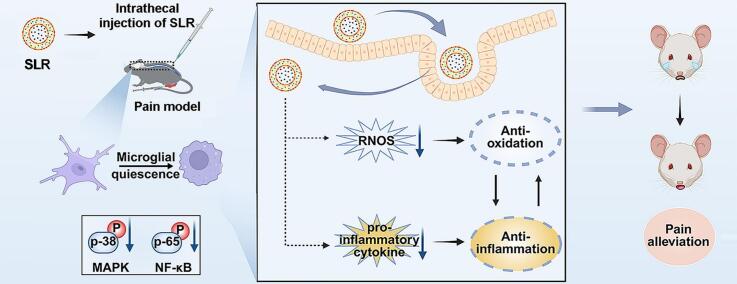

## 1 Introduction

Inflammatory pain, a debilitating form of chronic pain, significantly compromises the quality of life for millions worldwide (Cao et al., 2024a; Cohen et al., 2021; Kidd and Urban, 2001). It begins with inflammatory mediators released at the site of tissue damage, which decreases nociceptor excitability thresholds to induce peripheral sensitization (Emery et al., 2012; Miura et al., 2011). This persistent sensory input activates spinal glial cells, triggering neuroinflammation. Glial-derived inflammatory molecules promote central sensitization. This process establishes chronic central nervous system (CNS) hyperexcitability, which amplifies pain signals. Consequently, patients experience clinical manifestations such as allodynia and hyperalgesia (Ji et al., 2018). Current clinical medications for chronic pain primarily include opioids and non-steroidal anti-inflammatory drugs (NSAIDs). However, these treatments are limited by their short duration of action and severe side effects. Common complications include gastrointestinal discomfort, hormonal disturbances, and the development of tolerance (Jiang et al., 2020; Woolf, 2020; Xu et al., 2023; Zhou et al., 2021). Therefore, exploring novel analgesic drugs with minimal adverse reactions and long-lasting effects is a crucial direction for future research (Akhilesh et al., 2025; Liu et al., 2024; Lu et al., 2024; Mohsin et al., 2025; Wedel et al., 2022).

Ongoing investigations have progressively elucidated the complex molecular landscape underlying chronic pain (Babu et al., 2024; Cao et al., 2024b; Lu et al., 2025; Modi et al., 2023). Notably, emerging evidence highlights the synergistic roles of neuroinflammation and oxidative stress in maintaining these persistent pain states (Ji et al., 2016; Kidd and Urban, 2001; Lin et al., 2022; Scholz, 2014; Wu et al., 2025). These two processes form a self-perpetuating vicious cycle: inflammatory signaling generates reactive nitrogen and oxygen species (RNOS), leading to oxidative stress, which in turn amplifies inflammatory signaling and perpetuates pain sensitization (Grace et al., 2016; Salvemini et al., 2011). Importantly, as highly dynamic immune cells of the CNS, microglia are actively involved in this process (Su et al., 2022; Tansley et al., 2022). Activated by the inflammatory environment, microglia produce RNOS, pro-inflammatory factors, and other neurotoxic substances, thereby further amplifying pain signals and aggravating pain (Choi et al., 2019). Thus, breaking this reciprocal feedback loop through dual-action antioxidant and anti-inflammatory intervention represents a highly motivated therapeutic strategy.

Nanozymes are nanomaterials with intrinsic enzyme-like catalytic properties (Gao et al., 2007). Their stability, cost-effectiveness, and tunability make them attractive alternatives or complements to natural enzymes across a rapidly expanding range of applications (Fan et al., 2024; Li et al., 2023; Zhang et al., 2024; Zhang et al., 2021). Recently, nanozymes with antioxidant enzyme-like activities (*e.g.*, superoxide dismutase (SOD) and glutathione peroxidase (GPx)) have been explored for treating oxidative stress-related diseases (Xu et al., 2024; Yang et al., 2024a, Yang et al., 2024b). However, current research into nanozyme-based pain alleviation remains limited. Most studies focus narrowly on reactive oxygen species (ROS) elimination, often overlooking the critical role of reactive nitrogen species (RNS) and the synergistic impact of inflammation on pain progression (Bai et al., 2024; Cheng et al., 2025; Ling et al., 2023; Mu et al., 2019; Zhao et al., 2025). Therefore, we hypothesize that combining anti-inflammatory agents with antioxidant nanozymes could offer a more comprehensive and effective treatment for chronic pain.

Among various nanozymes, carbon quantum dots (CQDs) have garnered significant interest due to their zero-dimensional structure and excellent biocompatibility (Wang et al., 2024; Zhang et al., 2025). By incorporating Selenium (Se), an essential trace element that maintains redox balance and inhibits apoptosis, Se-doped carbon quantum dots (SeCQDs) emerge as a novel therapeutic platform for oxidative stress (Li et al., 2017). Despite their potential, the application of SeCQDs in pain relief remains largely unexplored. Furthermore, their ultrasmall size results in rapid metabolism. This metabolic rate hinders the achievement of long-lasting analgesic effects. On the other hand, Raddeanin A (RA) is a triterpenoid isolated from the traditional Chinese medicinal herb Anemone raddeana Regel. While it possesses potent anti-inflammatory properties, it suffers from poor bioavailability. This is primarily due to its high molecular weight and hydrophobicity (Hei et al., 2022; Wang et al., 2018).

**Scheme 1:**
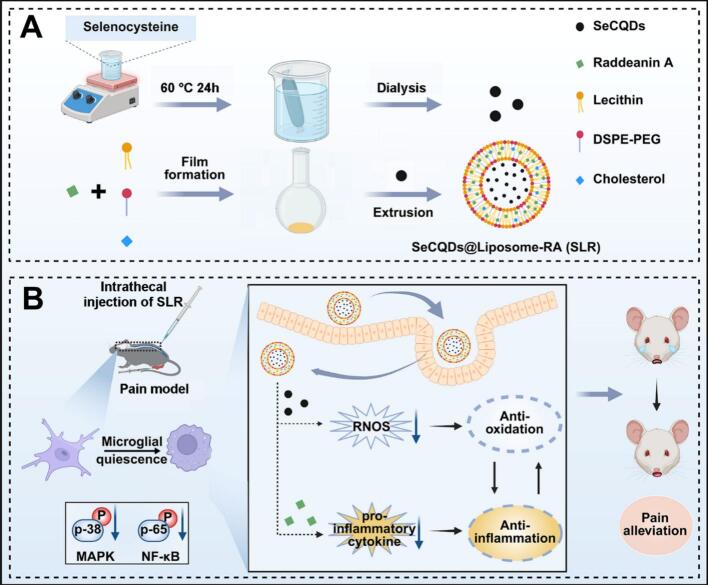
Schematic diagram of (A) the synthesis route of SLR nanoparticles and (B) the use of SLR for chronic pain with synergistic antioxidant and anti-inflammatory effects and their molecular analgesic mechanism.

Herein, we developed a liposome-based assembly strategy for the co-delivery of SeCQDs and RA, aiming to alleviate chronic pain with long-lasting effects through their synergistic antioxidant and anti-inflammatory actions. SeCQDs@Liposome-RA (SLR) nanoparticles were synthesized *via* extrusion method, effectively embedding hydrophobic RA within the liposome membrane and encapsulating hydrophilic SeCQDs within its cavity (Scheme 1A). In activated microglia, SLR nanoparticles significantly scavenged free radicals and reduced inflammatory cytokines. These activities effectively inhibited the mitogen-activated protein kinase (MAPK)/nuclear factor kappa B (NF-κB) signaling pathways, thereby reversing the activation of microglia (Scheme 1B). Crucially, bolstered by the liposomes' sustained-release capabilities, SLR nanoparticles provided analgesia in a Complete Freund's Adjuvant (CFA)-induced inflammatory pain model for up to 72 h. Furthermore, SLR exhibited robust analgesic efficiency in both chemotherapy-induced neuropathic pain (CIPN) and spared nerve injury (SNI)-induced neuropathic pain model. Overall, this therapeutic strategy substantially extended the duration of analgesic effects, presenting a promising approach for treating chroinc pain.

## 2 Materials and methods

### 2.1 Materials

L-selenocystine, lecithin, DSPE-PEG, cholesterol, raddeanin A were obtained from Shanghai Macklin Biochemical Technology Co., Ltd. Dulbecco's Modified Eagle Medium (DMEM) and DF-12 medium were supplied by Shanghai Yuanpei Biotech Co., Ltd. The RNA extraction kit was purchased from Shanghai Yishan Biotechnology Co., Ltd. The RNA reverse transcription kit, quantitative polymerase chain reaction (q-PCR) kit, PCR centrifuge tubes, q-PCR plates, and polyvinylidene fluoride (PVDF) membranes were obtained from Vazyme Biotechnology Co., Ltd. Primary antibodies against phosphorylated p38 (cat. no. 81212–2-RR), p38 (cat. no. 80821-2RR), p65 (cat. no. 10745–1-AP), phosphorylated JNK (cat. no. 80024–1-RR), JNK (cat. no. 81629–1-RR), phosphorylated ERK (cat. no. 80031–1-RR), ERK (cat. no. 83533–1-RR), phosphorylated IκBα (cat. no. 82349–1-RR), IκBα (cat. no. 80019–1-RR), as well as the HRP labeled secondary antibody (cat. no. RGAR001) were purchased from Proteintech Group, Inc. The phosphorylated p65 antibody (cat. no. 3033) was obtained from Cell Signaling Technology. The ionized calcium-binding adapter molecule 1 (IBA-1) primary antibody (cat. no. YM8165) was purchased from Immunoway. GAPDH primary antibody (cat. no. BM1623), HRP labeled secondary antibody (cat. no. BA1062) and DyLight 488-conjugated AffiniPure Donkey Anti-Rabbit IgG (cat. no. BA1146) were acquired from Wuhan Boster Biological Technology, Ltd. The hematoxylin and eosin (H&E) staining kit and Hoechst was purchased from Beyotime Institute of Biotechnology (Beijing, China).

### 2.2 Synthesis of SeCQDs

200 mg of selenocysteine was added to 10 mL of deionized water and the pH of the solution was adjusted to approximately 9 using a 0.5 M NaOH solution. The solution was heated at 60 °C for 24 h, followed by centrifugation (14,750 *g*, 15 min). The supernatant was collected and dialyzed (MWCO 500 Da) for 2 days. The concentration of the SeCQDs was determined by measuring the Se content *via* inductively coupled plasma-optical emission spectrometer (ICP-OES), which was then used as the SeCQD concentration.

### 2.3 Synthesis of SLR

Lecithin, cholesterol, and DSPE-PEG2K (molar ratio 52:30:3) were dissolved in chloroform, and the solution was mixed with RA dissolved in methanol (molar ratio 15). The solvent was removed by static volatilization to form a lipid film. The film was hydrated with 1 mL of a SeCQDs aqueous solution, followed by ultrasonic treatment (800 W, 30 s). The resulting emulsion was then extruded (100 nm membrane) to produce liposomes. SLR was purified by dialysis (MWCO 3500 Da) for 2 days.

### 2.4 Characterization of SeCQDs and SLR

The physicochemical properties of SeCQDs and SLR were comprehensively characterized. Morphology and particle size were visualized *via* transmission electron microscopy (TEM). Samples were ultrasonicated for uniform dispersion, deposited onto carbon-coated copper grids, and, in the case of SLR, negatively stained with 1% phosphotungstic acid for 3 min. After air-drying, specimens were imaged to assess internal structure. Dynamic light scattering (DLS) using a Malvern Zetasizer determined the hydrodynamic size, polydispersity index (PDI), and zeta potential of the nanoparticles following sonication in deionized water. Atomic force microscopy (AFM) was employed to measure SeCQD thickness, while X-ray photoelectron spectroscopy (XPS) identified elemental compositions and valence states. Optical properties were recorded using Shimadzu UV–vis and Prism fluorescence spectrophotometers. The encapsulation efficiency of RA in SLR was determined by high-performance liquid chromatography (HPLC) using an acetonitrile-water mobile phase (65:35, containing 0.05% glacial acetic acid) at a flow rate of 1 mL/min and detection at 205 nm (UV detector). And the encapsulation efficiency of SeCQDs was determined by ICP-OES.

### 2.5 Release profile of SLR *in vitro*

A volume of 1 mL of the 160 μg/mL SLR solution was pipetted and mixed with 9 mL of PBS buffer to prepare a 10 mL incubation system, which was then continuously shaken at 100 rpm in a thermostatic shaker at 37 °C. At the predetermined time points (0, 4, 8, 12, 24, 48, 72, 96, and 120 h), 500 μL of the incubation solution was withdrawn, and an equal volume of fresh PBS buffer was immediately replenished to maintain a constant system volume. The withdrawn incubation solution was separated using a centrifugal filter with a MWCO of 5000 to retain the unreleased SLR carriers. The free RA in the filtrate was collected, and its cumulative release amount was determined and calculated *via* HPLC.

### 2.6 Antioxidant properties of SeCQDs and SLR

The antioxidant enzyme-like activities of SeCQDs and SLR were evaluated using UV–vis spectrophotometry. For all assays, a series of concentration gradients for both SeCQDs and SLR were prepared in a total reaction volume of 500 μL. The glutathione peroxidase (GPx)-like activity was measured by monitoring the oxidation of NADPH. The reaction mixture contained GSH (2 mM), NADPH (0.4 mM), glutathione reductase (0.85 U/mL), H_2_O_2_ (240 μM) and PBS (pH 7.4, 2.5 mM). After assembly, the decrease in absorbance at 340 nm was recorded over 300 s in dynamic mode. The superoxide dismutase (SOD)-like activity was assessed using the riboflavin-methionine-NBT system. The mixture consisted of methionine (10 mM), riboflavin (20 μM), NBT (0.1 μM), and PBS (7.4, 5 mM). Following a 5-min incubation at room temperature under light, the absorbance was measured across a wavelength range of 400–800 nm. The hydroxyl radical scavenging ability was determined using methylene blue (MB) assay. The system comprised FeSO_4_ (1 mM), H_2_O_2_ (0.5 mM), and MB (0.025 mM). After incubation at room temperature for 15 min, the absorbance was recorded between 500 and 800 nm.

The total antioxidant capacity was assessed through 2,2′-azino-bis(3-ethylbenzothiazoline-6-sulfonic acid) (ABTS) and 2,2-diphenyl-1-picrylhydrazyl (DPPH) radical scavenging assays. The ABTS working solution was prepared by mixing 5 mL of 7.4 mM ABTS with 88 μL of 2.6 mM K₂S₂O₈ solution and storing the mixture in the dark at room temperature for 16 h. This stock solution was diluted with water until its absorbance at 734 nm was ≈0.70. To determine the ABTS free radical scavenging activity, 100 μL of the sample was mixed with 400 μL of the diluted ABTS solution. After a 12-h incubation, the absorbance (A) was read at 734 nm. For the DPPH assay, 100 μL of the sample was reacted with 400 μL of a 100 μM DPPH-ethanol solution. The reaction proceeded for 12 h in the dark at room temperature, followed by absorbance measurement at 517 nm (A) using a spectrophotometer. For both assays, the radical scavenging activity was calculated using the following equation:

$$\text{Scavenging activity}\;\left(\%\right)=\frac{A_{0}-A}{A_{0}}\times 100\%$$

where A_0_ represents the absorbance of the control (mixture without sample) and A represents the absorbance of the sample.

### 2.7 Cell culture

The BV2 cell line was acquired from the Institute of Biochemistry and Cell Biology, Chinese Academy of Sciences (CAS Institute), Shanghai, China. BV2 cells were cultured in DMEM medium supplemented with 10% fetal bovine serum, 1% penicillin/streptomycin solution in a cell culture incubator.

Primary microglial and astrocyte cultures were established from the cerebral tissues of neonatal mice (1–3 days old). Brain tissues were dissociated with 0.05% trypsin to generate single-cell suspensions. For microglial enrichment, cells were resuspended in DMEM/F-12 complete medium supplemented with 5 ng/mL GM-CSF, 10% fetal bovine serum (FBS), 1% penicillin/streptomycin, and 1% glutamine. After 14 days of incubation at 37 °C with 5% CO_2_, floating microglia were harvested from the supernatant and transferred to GM-CSF-free medium for a 24-h resting period. Conversely, astrocytes were cultured in DMEM/F-12 medium supplemented with 10% FBS, 1% penicillin/streptomycin, and 1% glutamine, but without GM-CSF, for 8–10 days. These astrocytes were subsequently transferred to fresh medium for at least 24 h of resting culture prior to experimental use. The neurons used in this study were kindly provided by Professor Wu Xiaomei's research group.

### 2.8 Cell cytotoxicity test

100 μL of cell suspension (BV2 cells, primary microglia, microglia or neurons) was cultured in a 96-well plate. After the cells adhered to the wall, they were used for the cytotoxicity test. Test materials with gradient concentrations were added to the 96-well plate and incubated. After 24 h, the cell viability was determined using a Cell Counting Kit-8 (CCK-8) assay.

### 2.9 Cellular uptake analysis

BV2 cells and primary microglia were seeded into confocal dishes. Once adhered, cells were treated with 4 μg/mL SLR-Cy5.5 in culture medium for 2 h. Control cells received medium only. After treatment, the medium was aspirated, and cells were washed twice with PBS before being fixed with 1 mL of 4% PFA for 15 min. The PFA was removed, and cells were washed again with PBS and counterstained with Hoechst for 10 min. Fluorescent signals were then observed using a confocal microscope.

### 2.10 Evaluation of intracellular RNOS

Cells (BV2 cells and primary microglia) were seeded in confocal culture dishes. Once adhered, cells were pre-incubated with the test materials for 2 h. Oxidative stress was then induced by adding 40 μM tert-butyl hydroperoxide (t-BOOH) for 1 h. A blank control group (no t-BOOH or test materials) was run simultaneously. Following treatment, all groups were stained with a 10 μM 2′,7′-dichlorofluorescein diacetate (DCFH-DA) probe for 30 min and a 10 μg/mL Hoechst solution for 5 min. RNOS production was assessed by measuring the DCFH-DA fluorescence intensity using a confocal microscope.

### 2.11 Pro-inflammatory factor mRNA levels quantified by RT-qPCR

BV2 cells were seeded in 6-well plates and, upon adherence, were stimulated with 100 ng/mL LPS and test nanomaterials (SeCQDs at 4 μg/mL, RA at 2 μg/mL, or SLR at 8 μg/mL) for 24 h before total RNA extraction. For primary microglia, cells were pre-incubated with nanomaterials (SeCQDs at 4 μg/mL, RA at 1 μg/mL, or SLR at 4 μg/mL) for 2 h. They were then stimulated with 10 ng/mL LPS (plus nanomaterials) for 3.5 h and finally activated with 10 μM Nigericin for 30 min before RNA extraction. For mice, the L4–6 spinal cords and cerebral cortex were dissected after respective treatments in different groups. Total RNA was extracted using a rapid RNA extraction kit. Next, 1 μg of total RNA was reverse transcribed into cDNA using a reverse transcription kit. In addition, qPCR analysis was performed using SYBR Green dye detection on a real-time detection system, and data analysis was conducted using the 2^-ΔΔCT^ method.

### 2.12 Western blotting

For cells, an appropriate amount of primary microglia was seeded in 6-well plates. After the cells adhered to the wall, nanomaterials (SLR, 4 μg/mL) were first added for incubation for 2 h. Then, the medium was replaced with a system containing nanomaterials and 10 ng/mL LPS, and incubation was continued for 4 h, after which the cells were collected. In addition, for groups treated with anisomycin, on this basis, the medium was replaced with a system containing nanomaterials, 10 ng/mL LPS and 3 μM anisomycin, and incubation was continued for 0.5 h, after which the cells were collected. For mice, the L4–6 spinal cords and cerebral cortex were dissected after respective treatments in different groups. Cells, spinal cords or cerebral cortex were homogenized by adding an appropriate amount of lysis buffer (containing protease and phosphatase inhibitors). The protein concentration of the protein supernatant was determined using the BCA protein assay. 30 μg of total protein was loaded per lane and separated on a 12% SDS-PAGE gel. After transfer and blocking with 5% BSA, the membranes were incubated with the corresponding primary antibodies (1: 8000 for p-p38 and p38; 1:1000 for p-p65 and p65; 1:3500 for p-JNK; 1:50000 for JNK; 1:5000 for p-ERK; 1:1000 for ERK; 1:3500 for p-IKBα and 1:5000 for IKBα) overnight at 4 °C. After washing, the membranes were incubated with the secondary antibodies for 1 h. Bands were imaged using the Odyssey system. Following detection of the phosphorylated protein, the membrane was stripped (Biosharp, BL1381B) and re-probed with the subsequent primary antibody. Protein quantification was performed using Image J.

### 2.13 Animals and Pain Models

Adult male ICR mice weighing approximately 24 g were supplied by the Experimental Animal Center of Nantong University (Nantong, China). Mice were housed in a controlled environment at 26 °C with a 12-h light/dark cycle and controlled humidity, with libitum access to standard food and water. All animal procedures were performed in accordance with the ARRIVE guidelines and the National Institutes of Health Guide for the Care and Use of Laboratory Animals. Ethical approval was obtained from the Experimental Animal Center of Nantong University (approval No. S20241216–002). All efforts were made to minimize animal suffering and to reduce the number of animals used. For the inflammatory pain model, mice were briefly anesthetized with isoflurane, and 20 μL of complete Freund's adjuvant (CFA) was injected subcutaneously into the plantar surface of the left hind paw. Successful induction was confirmed by the presence of paw swelling, licking, and stable thermal hyperalgesia and mechanical allodynia at 3 days post-injection. For the chemotherapy-induced peripheral neuropathy (CIPN) model, paclitaxel was dissolved in a vehicle of 50% Cremophor EL and 50% anhydrous ethanol to prepare a 6 mg/mL stock solution, which was further diluted with normal saline to 0.2 mg/mL immediately before administration. Mice received intraperitoneal injections (i.p.) of paclitaxel at 2 mg/kg daily for 5 consecutive days, resulting in a cumulative dose of 10 mg/kg. Painful neuropathy and thermal hyperalgesia were reliably established on day 7 after the first paclitaxel injection. For the spared nerve injury (SNI) model, mice were anesthetized, and a 1–1.5 cm longitudinal incision was made along the femur on the lateral side of the left thigh. Following blunt dissection of skin and subcutaneous tissue, the biceps femoris muscle was separated to expose the sciatic nerve. The nerve was traced distally to identify the tibial, common personnel, and sural branches. The common peroneal and sural nerves were carefully isolated using micro-ophthalmic forceps and ligated with silk sutures approximately 2 mm distal to the nerve bifurcation. The muscle and skin layers were closed sequentially, and behavioral testing was performed 7 days after surgery.

### 2.14 Biodistribution of nanoparticles in mice

CFA-model mice were briefly anesthetized with inhalational anesthesia and placed in a prone position for *in vivo* fluorescence imaging using a small-animal imaging system. Serial whole-body images were acquired at 0 h, 5 min, 0.5 h, 1 h, 2 h, 4 h, 8 h, 12 h, 24 h, 36 h, 48 h, 60 h, 72 h, 84 h, 96 h, 108 h, and 120 h following intrathecal (i.t., *n* = 6) or intravenous (i.v., n = 6) administration of SLR-Cy5.5. For *ex vivo* imaging, heart, liver, spleen, lung, kidney, brain, and spinal cord tissues were harvested at 0, 24, 72, and 120 h and immediately scanned using the same imaging system.

### 2.15 Behavioral tests

Mice were acclimated to testing chambers on an elevated metal mesh floor for at least 2 consecutive days, 30 min per day, before baseline testing to minimize stress-related bias. For mechanical nociception, the von Frey test was performed using a set of calibrated von Frey filaments with logarithmically increasing stiffness. The 50% paw withdrawal threshold (PWT) was calculated using the Dixon up-down method, with PWT presented as the primary behavioral outcome. For thermal nociception, the Hargreaves test was used. An infrared radiant heat source was positioned beneath the plantar surface of the hind paw, and the paw withdrawal latency was recorded as the time to first nociceptive response. All behavioral tests were conducted by experimenters blinded to group allocation to avoid observer bias. Body weight was monitored throughout the experimental period to assess general health and potential treatment-related toxicity.

For mice with the CFA inflammatory pain model, baseline mechanical and thermal nociceptive thresholds were measured in mice prior to the induction of the CFA-induced inflammatory pain model. This model was established on Day −4 (4 days before SLR administration). Three days later (Day −1), nociceptive thresholds were re-assessed to confirm the development of inflammatory pain. On Day 0, mice were randomly assigned to groups (*n* = 5 per group) and administered with SLR (67 μg/kg, i.t.), RA (150 μg/kg, i.t.), liposome (1 mg/kg, i.t.), SeCQDs (67 μg/kg, i.t.), ibuprofen (30 mg/kg, i.p.), or PBS. After that, mechanical and thermal nociceptive thresholds were dynamically monitored and recorded at 2, 4, 8, 12, 24, 48, 72, 96, and 120 h post-administration. To evaluate the effect of other injection ways (i.v. injection and intraplantar (i.pl.) injection) on pain-relieving effect of SLR nanoparticles, SLR nanoparticles (0.2 mg/kg for i.pl. and 1 mg/kg for i.v.) or PBS were injected into CFA-model mice, with the mechanical and thermal nociceptive thresholds assessed at the same time intervals.

For mice with the CIPN model, baseline mechanical and thermal nociceptive thresholds were first measured. The CIPN model was established from day −7 to day −2 (7 to 2 days before administration), and nociceptive thresholds were re-assessed on day −1 (1 day before administration / 1 day after modeling). On Day 0, mice were randomly assigned to groups (n = 5 per group) and administered with SLR (67 μg/kg, i.t.) or PBS. After that, mechanical and thermal nociceptive thresholds were dynamically monitored and recorded at 2, 4, 8, 12, 24, 48, and 72 h post-administration.

For mice with the SNI neuropathic pain model, baseline mechanical and thermal nociceptive thresholds were first measured. The SNI model was established on Day −7 (7 days before SLR administration), and nociceptive thresholds were re-tested on Day −1 (1 day before administration/6 days after modeling). On Day 0, mice were randomly assigned to groups (n = 5 per group) and administered with SLR (67 μg/kg, i.t.) or PBS. After that, thresholds were dynamically monitored and recorded at 2, 4, 8, 12, 24, and 48 h post-administration.

### 2.16 Assessment of spinal cord oxidative stress

The L4-L6 spinal cord segments were dissected and fixed in 4% paraformaldehyde (PFA) at 4 °C overnight. Tissues were then cryoprotected in graded sucrose solutions (20% followed by 30%) until sinking. Following frozen embedding and sectioning, oxidative fluorescent staining was performed using a dihydroethidium (DHE) kit, and images were captured under a fluorescence microscope.

### 2.17 Immunohistochemistry

L4-L6 spinal cord segments were harvested and fixed in 4% PFA at 4 °C overnight, then dehydrated in 20% and 30% sucrose solutions. After cryo-embedding and sectioning, sections were blocked with 10% BSA in PBST for 2 h, then incubated with primary antibodies (IBA-1, 1:4000) at 4 °C overnight. After PBST washing, sections were incubated with Dylight 488-conjugated secondary antibodies at room temperature for 2 h, and immunofluorescence was visualized using a fluorescence microscope.

### 2.18 *In vivo* biosafety assessment

To evaluate the long-term neurotoxicity and locomotor effects of the treatment, male ICR mice (*n* = 8 per group) were administered a single i.t. injection of SLR (67 μg kg^−1^). After 15 days, mice underwent a battery of behavioral assessments, including the open-field test (OFT) and the novel object recognition (NOR) experiment. For the OFT, individual mice was placed in a 50 cm × 50 cm × 50 cm white polyvinyl chloride box and allowed to move freely for 5 min. An overhead camera was employed to record and analyze the movement trajectory and time spent in the peripheral (25 cm from the wall) and central zones (25 cm × 25 cm). For the NOR, a 50 cm × 50 cm × 50 cm square arena contained two identical objects (A and B, 3 cm diameter) symmetrically placed. Mice were gently introduced facing the opposite wall, and exploration was defined as nose contact within 3 cm of an object. During the training phase, exploration time for both objects was recorded over 5 min. After a 12 h interval, object B was replaced with novel object C (similar size but distinct shape) for the test phase, and exploration time for objects A and C was measured. The discrimination index was calculated to assess recognition memory by using the following equation:

$$\text{Discrimination index}\;\left(\%\right)=\frac{\mathrm{TC}}{\mathrm{TA}+\mathrm{TC}}\times 100\%$$

where TA represents exploration time for object A and TC represents exploration time for object C.

In addition, 15 days after i.t. injection of SLR (67 μg kg^−1^), the mice were sacrificed. The blood IgG level in blood was tested *via* ELISA (Solarbio, SEKM-0098). *Tnfa* and *Il1b* expression in the spinal cord and cerebral cortex were quantified using RT-qPCR. The main organs, including the heart, liver, spleen, lungs, kidneys and brain, were taken out, photographed and compared with those of normal mice, and H&E staining was performed. Meanwhile, another batch of mice (*n* = 8) was sacrificed 30 days after injection, and blood was collected to obtain blood routine and serum biochemical index data.

### 2.19 Statistical analysis

Data processing and image analysis in this study were performed using Origin 2021, GraphPad Prism, Excel, Nano Measurer 1.2, Avantage, Gwyddion, and Image J software. Quantitative results are presented as mean ± standard deviation (mean ± SD) or mean ± standard error of the mean (mean ± SEM). Student's *t*-test was used for comparisons between two groups and one-way ANOVA followed by Bonferroni's *post hoc* test was used for comparisons among multiple groups. For behavioral experiments with repeated measures, two-way mixed ANOVA followed by Tukey's post-hoc test was used. Differences were considered statistically significant at *p* < 0.05 (*), *p* < 0.01(**) *p* < 0.001 (***), as well as p < 0.05 (#), p < 0.01 (##), p < 0.001 (###). Nonsignificant differences were labeled “n.s.”

## 3 Results and discussion

### 3.1 Synthesis and characterization of SeCQDs

**Fig. 1:**
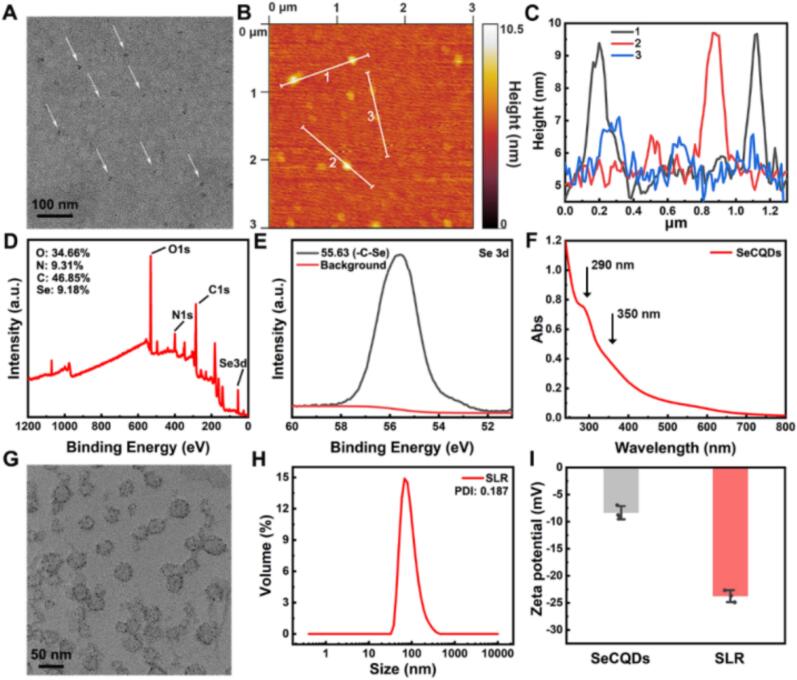
Structural and compositional characterization of SeCQDs and SLR nanoparticles. (A) TEM image of SeCQDs. (B) AFM image of SeCQDs and (C) the corresponding height profile along lines 1, 2, and 3. (D) XPS spectrum of SeCQDs and (E) high-resolution spectra of Se 3d. (F) UV–Vis absorption spectrum of SeCQDs. (G) TEM image and (H) hydrodynamic diameter distribution of SLR. (I) Zeta potential plots of SeCQDs and SLR (*n* = 3).

Prior to the preparation of SLR nanoparticles, the SeCQDs were synthesized *via* a hydrothermal method by treating selenocystine solution, according to the previous report. The morphology and size of the synthesized SeCQDs were characterized by transmission electron microscopy (TEM). As depicted in Fig. 1A, the SeCQDs exhibited a uniform distribution with an average diameter of 5.8 nm, a result corroborated by atomic force microscopy (AFM) measurements showing topographic heights of approximately 1–6 nm (Fig. 1B and C). The elements and the valence state of SeCQDs were analyzed by the X-ray photoelectron spectroscopy (XPS). Based on the results (Fig. 1D), the nanocomposites contained C, N, O and Se elements as there were the corresponding peaks of C 1 s, N 1 s, O 1 s, Se 3d. And corresponding content of these elements was inserted in Fig. 1D. The high resolution XPS spectrum of C 1 s (Fig. S1) exhibited peaks at the binding energy of 284.8, 286.2 and 287.9 eV, which corresponded to the C-C, C-O/C-Se/C-N and C=O, respectively. The high-resolution N 1 s spectrum (Fig. S2) showed two peaks at 399.4 and 401.6 eV, corresponding to pyridinic-N and pyrrolic-N, respectively. Furthermore, the Se 3d spectrum (Fig. 1E) revealed the presence of Se-C in the nanoparticles. The results confirmed that Se was embedded into the SeCQDs. In addition, the UV–Vis absorption spectrum of SeCQDs was further tested (Fig. 1F). Two bands at around 290 nm and 350 nm were detected, which were attributed to π-π transition of the C-C bond and n-π transitions of C-O bonds or other surface groups in conjugated sp^2^ domains, respectively. Finally, the fluorescent characteristic of SeCQDs was further tested (Fig. S3). The fluorescence spectra showed primary excitation and emission peaks at 410 and 486 nm, respectively. All the above results proved the successful synthesis of SeCQDs.

### 3.2 Synthesis and characterization of SLR

After that, SLR nanoparticles were synthesized *via* extrusion method. In detail, RA was mixed with 1,2-distearoyl-sn-glycero-3-phosphoethanolamine-PEG, lecithin, cholesterol and the main components of liposome. After volatilization into a film, SeCQDs solution was added and the SLR nanoparticles were obtained by extrusion method. The obtained SLR nanoparticles were characterized by TEM (Fig. 1G), which were nanosized round vesicels with dots distributed on it. It proved the successful encapsulation of SeCQDs in SLR nanoparticles. In addition, the particle size of SLR was evaluated by dynamic light scattering (DLS), which showed diameter around 111.1 nm (Fig. 1H). And the zeta potential of SLR was −23.5 mV, slightly negative than SeCQDs (Fig. 1I). After that, the encapsulation rate of RA and SeCQDs in SLR was tested by high performance liquid chromatography (HPLC) and inductively coupled plasma-optical emission spectrometer (ICP-OES), respectively. The results (Fig. S4) showed the encapsulation efficiency of RA was around 22.2% and the one of SeCQDs was 70.7%. These results illustrated the successful preparation of SLR nanoparticles. Subsequently, the *in vitro* release profile of RA from SLR was evaluated in PBS at 37 °C using HPLC. As shown in Fig. S5, SLR exhibited sustained-release kinetics, confirming its potential as a long-acting drug delivery system.

### 3.3 RNOS elimination properties of SeCQDs and SLR

Next, the antioxidant properties of SeCQDs were investigated. Given that Se is a core component of GPx, a key enzyme that detoxifies H_2_O_2_ by oxidizing GSH to GSSG, the GPx-like activity of SeCQDs was investigated first. To assess GPx-like activity, we coupled this reaction with glutathione reductase, which regenerates GSH from GSSG while consuming NADPH (Fig. S6A). The real-time change in NADPH's UV–Vis absorption at 340 nm was subsequently monitored to reflect the kinetics of these coupled reactions. As shown in Fig. S6B, an obvious decrease of NADPH concentration was observed with all components present, and this reduction intensified as the SeCQD concentration increased. The result indicated the SeCQDs had high GPx-like enzyme activity to remove excess H_2_O_2_. SOD was another significant antioxidant enzyme known to catalyze the disproportionation of O_2_^•-^. The SOD-like activity of SeCQDs was further studied by utilizing an assay that measures their inhibition of nitrotetrazolium blue chloride (NBT) photoreduction (Fig. S7A). Since O_2_^•-^ reduces NBT to generate blue formazan under UV irradiation, a decrease in blue color indicates O_2_^•-^ scavenging. The results (Fig. S7B) clearly showed that SeCQDs effectively scavenged the generated O_2_^•-^ *via* an SOD-like enzyme catalysis pathway. In addition, the •OH elimination ability of SeCQDs was further studied. We monitored this by using methylene blue (MB) as indicator as it can be degraded by •OH with a color change (Fig. S8A). The results (Fig. S8B) illustrated that the SeCQDs exhibited •OH scavenging capability, with this ability demonstrating a concentration-dependent increase.

**Fig. 2:**
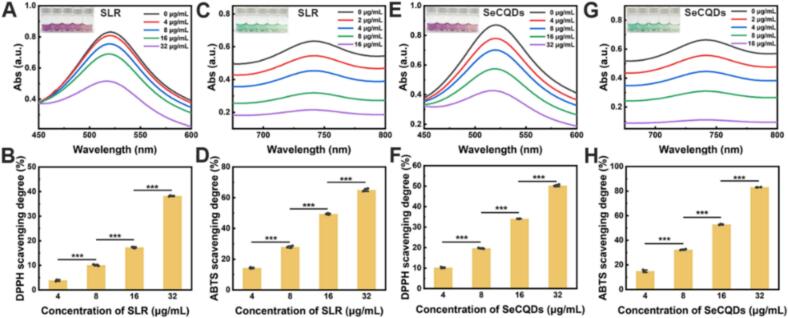
Radical elimination properties of SLR and SeCQDs. UV–Vis absorption spectra showing the scavenging effect of various concentrations of SLR and SeCQDs on DPPH (A, E) and ABTS (C, G) free radicals. The corresponding scavenging percentages for DPPH (B, F) and ABTS (D, H) were shown as a function of concentration. All data were means ± SD; *n* = 3. Statistical significance (*P < 0.05, **P < 0.01, ***P < 0.001) was determined by one-way ANOVA followed by Bonferroni's *post hoc* test.

The radical scavenging properties of SLR were evaluated against SeCQDs control. The 2,2-diphenyl-1-picrylhydrazyl (DPPH) assay was performed to assess SLR's capacity to scavenge RNS, revealing excellent antioxidant properties (Fig. 2A and B). We then used the 2,2′-azino-bis(3-ethylbenzothiazoline-6-sulfonic acid) (ABTS) assay to evaluate the total scavenging ability against RNOS. SLR exhibited a concentration-dependent RNOS scavenging ability (Fig. 2C and D). However, it was weaker than the SeCQDs (Fig. 2E-H). We hypothesized that the liposomal encapsulation of SLR hindered the interaction between the substrate and the active site, thereby reducing its overall RNOS scavenging efficacy.

### 3.4 Cellular cytotoxicity and internalization of SLR

**Fig. 3:**
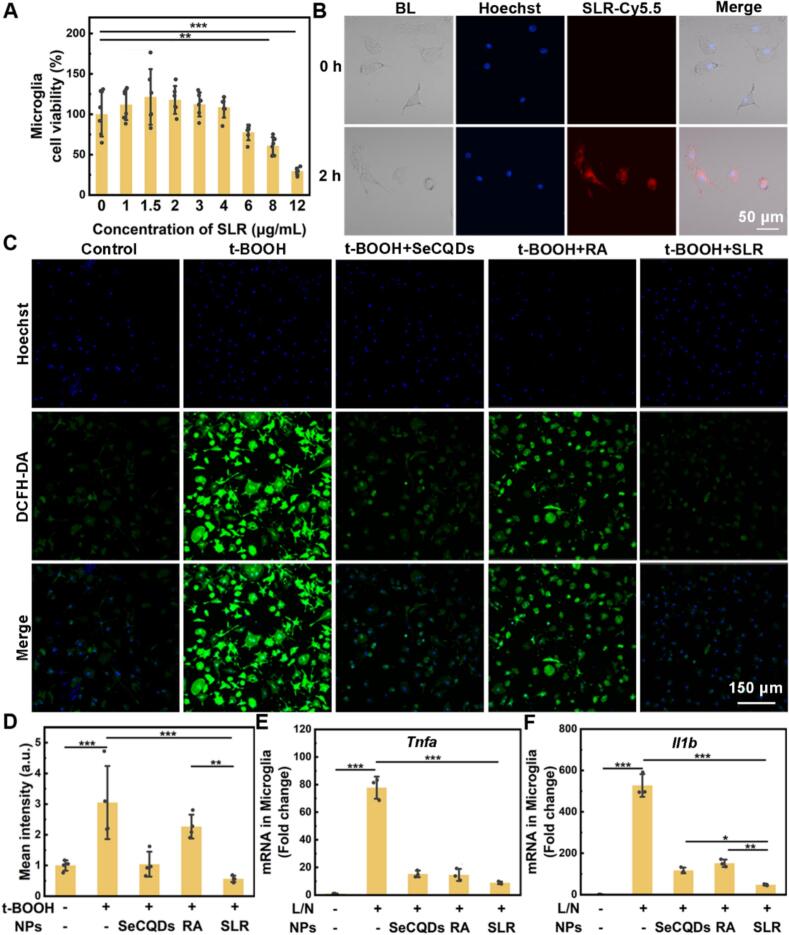
*In vitro* effects of SLR nanoparticles on primary microglia. (A) Cell viability of primary microglia treated with SLR nanoparticles at different concentrations (*n* = 6). (B) Fluorescence microscopy images of primary microglia incubated with SLR-Cy5.5 nanoparticles. (C) Confocal microscopy images illustrating the reduction of RNOS in t-BOOH-stimulated primary microglia following various treatments, as detected by the DCFH-DA probe. (D) Quantitative analysis of the mean DCFH-DA fluorescence intensity corresponding to RNOS levels (*n* = 4). RT-qPCR analysis of nanoparticle effects on TNF-α (E) and IL-1β (F) mRNA expression in LPS/nigericin (L/N)-stimulated primary microglia. (*n* = 3) All data are means ± SD. Statistical significance (*P < 0.05, **P < 0.01, ***P < 0.001) was determined by one-way ANOVA followed by Bonferroni's *post hoc* test.

Prior to studying the therapeutic effect of SLR, the cytotoxicity of SLR on microglia was tested first. Microglial cell line BV2 and primary microglia were used to incubate with varying concentrations of SLR, ranging from 0 to 12 μg/mL for 24 h. The result of cell counting kit (CCK-8) assay showed that SLR had no significant cytotoxicity against BV2 cells at concentrations below 8 μg/mL (Fig. S9) and against primary microglia at concentrations below 4 μg/mL (Fig. 3A). In contrast, both SeCQDs nanoparticles and RA alone exhibited higher cytotoxicity (Fig. S10 and S11). These results indicated that the liposome significantly improved the biocompatibility of the SeCQDs nanoparticles and RA. We also assessed SLR cytotoxicity in primary astrocytes and neurons, the two other major cell types in the nervous system. The observed toxicity was comparable to that of microglia, further confirming the nanoparticles' good biocompatibility (Fig. S12). Furthermore, fluorescence imaging was used to study the internalization of SLR in microglia. To track SLR, it was labeled with the Cy5.5 fluorescent dye using DSPE-PEG-Cy5.5, creating SLR-Cy5.5. After a two-hour incubation, the Cy5.5 signal was observed in the cytoplasm of both BV2 cells (Fig. S13) and primary microglia (Fig. 3B), indicating that SLR was successfully internalized by the cells.

### 3.5 Oxidative stress scavenging and inflammation reduction effects of SLR

Microglial cells are recognized as central modulators of pain perception. RNOS-induced oxidative stress triggers microglial activation, creating a positive feedback loop where activated microglia generate additional RNOS (Lu et al., 2021). This cycle plays a crucial role in the development and persistence of pain. The influence of SLR on intracellular RNOS levels was investigated by treating BV2 and primary microglia cells with tert-butyl hydroperoxide (t-BOOH) in the absence and presence of SLR. 2′,7′-dichlorofluorescein diacetate (DCFH-DA) was utilized as a fluorescent probe to quantify the RNOS concentration. The results of confocal microscopy revealed that SLR nanoparticles could effectively attenuate the t-BOOH-induced elevation in RNOS levels (Fig. 3C, D and S14). More importantly, SLR demonstrated a much higher RNOS scavenging ability compared to SeCQDs and RA alone, suggesting a synergistic antioxidant effect.

Furthermore, the influence of SLR on inflammatory factors of microglial cells was tested. Primary microglia cells were exposed to lipopolysaccharide (LPS) along with nigericin, either alone or with various components. After that, the inflammatory factors were analyzed using reverse transcription quantitative polymerase chain reaction (RT-qPCR) in cells. The results (Fig. 3E and F) indicated that LPS and nigericin stimulation markedly upregulated the expression of inflammatory mediators tumor necrosis factor alpha (*Tnfa*) and interleukin 1 beta (*Il1b*). However, SLR treatment significantly suppressed the expression of both *Tnfa* and *Il1b* to levels comparable to the LPS-untreated control. This reduction was notably greater than that achieved with either SeCQDs or RA alone, providing evidence of SLR's synergistic anti-inflammatory effect. Apart from that, similar experiments were performed on BV2 cells, and similar results were observed (Fig. S15). The consistent findings across both cell types collectively demonstrate that SLR possesses strong oxidative stress-scavenging and anti-inflammatory properties.

### 3.6 Mechanism of SLR in alleviating microglial inflammation

**Fig. 4:**
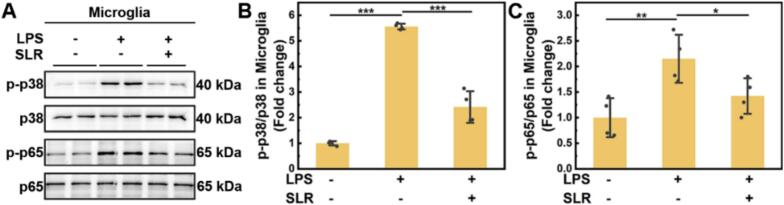
Mechanism of SLR nanoparticles in alleviating microglial inflammation. (A) Western blot analysis of p38 and p65 phosphorylation levels across different treatment groups. Quantitative analysis of the p-p38/p38 (B) and p-p65/p65 (C) protein expression ratios from panel A. All data are means ± SD; *n* = 4. Statistical significance (*P < 0.05, **P < 0.01, ***P < 0.001) was determined by one-way ANOVA followed by Bonferroni's *post hoc* test.

Based on the above results, we further tried to explore the mechanism of SLR in alleviating microglial inflammation. Previous studies have identified the MAPK/NF-κB signaling axis as a critical interface between inflammation and oxidant stress. Mechanistically, RNOS induces apoptosis signal-regulating kinase 1, which subsequently activates the p38/MAPK pathway to facilitate the expression of pro-inflammatory cytokines (Yuan et al., 2021). Simultaneously, NF-κB signaling orchestrates the rapid release of these mediators, fueling a self-perpetuating cycle of microglial activation (Chen et al., 2021; Liu et al., 2017). Given the antioxidant and anti-inflammatory properties of SLR, we hypothesized that it attenuates microglial neuroinflammation by modulating these dual signaling pathways. To verify it, western blot analysis was performed to assess the expression of phosphorylated p38 (p-p38) and p65 (p-p65) in primary microglia under various stimuli. The results (Fig. 4A-C, Fig. S16) demonstrated that the ratios of p-p38/p38 and p-p65/p65 were markedly elevated in LPS-treated cells compared to untreated controls. This increase, however, was suppressed by SLR nanoparticle treatment. Furthermore, ERK and JNK are also important protein kinases in MAPK pathways, while phosphorylation and subsequent degradation of IκBα are critical for NF-κB activation. We therefore examined the phosphorylation levels of ERK, JNK, and IκBα. The results indicated that SLR treatment also downregulated the LPS-induced phosphorylation of ERK, JNK, and IκBα (Fig. S17). To further confirm the involvement of the p38 MAPK pathway, we employed anisomycin, a canonical p38 activator that can also indirectly induce NF-κB activation. The results showed that anisomycin treatment significantly increased the p-p38/p38 ratio compared with the LPS + SLR group, effectively reversing the SLR-mediated inhibition of p38 phosphorylation (Fig. S18). Collectively, these findings suggested that SLR nanoparticles alleviate microglial inflammation by suppressing the activation of MAPK/NF-κB signaling pathways.

### 3.7 Biodistribution of SLR in a mouse model of CFA-induced inflammatory pain

**Fig. 5:**
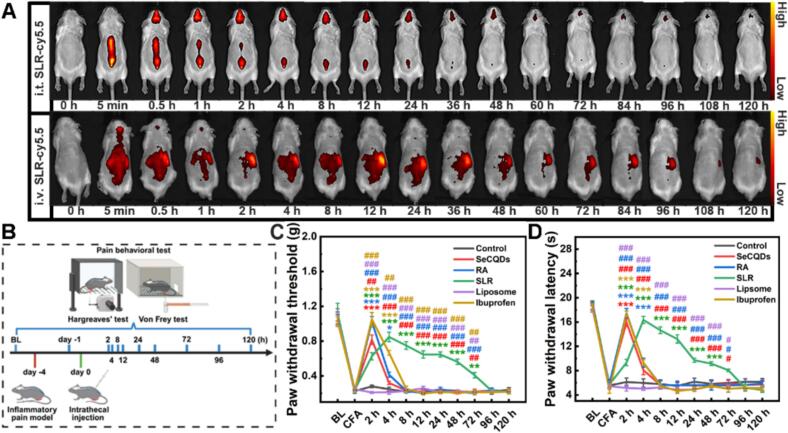
The biodistribution and analgesic efficacy of SLR nanoparticles in the CFA-induced inflammatory pain model. (A) *In vivo* fluorescence images of mice over time following i.t. and i.v. injection of SLR-Cy5.5 nanoparticles. (B) Schematic illustration of the CFA-induced inflammatory pain model and behavioral testing. Analgesic effects of i.t. administration of various nanoparticles on mechanical allodynia (C) and thermal hyperalgesia (D) in CFA-mice. (*n* = 5). Lines of different colors represent different groups. Asterisks (*) indicate comparison with the Control group. Hashtags (#) indicate comparison with the SLR group. All data are means ± SEM; *n* = 5. Statistical significance (*P < 0.05, **P < 0.01, ***P < 0.001) was determined by two-way mixed ANOVA followed by Tukey's post-hoc test.

Motivated by the above results, we explored the therapeutic effect of SLR on an inflammatory pain mouse model. The CFA-induced inflammatory pain model, which was widely recognized for its significant nociceptive and inflammatory characteristics, was used in our experiments to screen for anti-inflammatory pain activity. Firstly, we tested the biodistribution and clearance of SLR in a mouse model of CFA-induced inflammatory pain. To label the nanoparticles with fluorescent dye, DSPE-PEG-Cy5.5 was utilized for synthesizing SLR-Cy5.5 nanoparticles. After injecting SLR-Cy5.5 nanoparticles into mice intrathecally and intravenously, the fluorescent images of mice were captured at various time points, respectively. As the results shown, following intrathecal (i.t.) injection, SLR nanoparticles exhibited rapid and widespread distribution in the central nervous system, spreading throughout the spinal cord and reaching the brain within 30 min. The spinal cord signal decreased gradually while the brain signals peaked at approximately 2 h before gradually declining (Fig. 5A). Conversely, intravenous (i.v.) administration led to significant accumulation of SLR nanoparticles in the liver and kidney, with only a small, transient signal detected in the brain (Fig. S19). These findings confirmed that i.t. injection was a highly efficient means for delivering SLR nanoparticles to the central nervous system, whereas systemic delivery was limited by the blood-brain barrier and rapid clearance by the reticuloendothelial system organs. Additionally, the gradual metabolism of the nanoparticles throughout the body (Fig. S20) indicated a favorable biosafety profile *in vivo*.

### 3.8 The effect of SLR on a mouse model of CFA-induced inflammatory pain

Subsequently, we assessed the analgesic efficacy of SLR on CFA-induced inflammatory pain mouse model. The experiment was performed according to the schematic (Fig. 5B). The gold-standard von Frey filament and Hargreaves tests were used to measure paw withdraw threshold (PWT) and paw withdraw latency (PWL), respectively, to quantify mechanical and thermal allodynia. Notably, compared with the PBS group, the mice had the same PWT and PWL after i.t. injection of SLR nanoparticles (Fig. S21), demonstrating the i.t. injection of SLR did not interfere with the motor coordination ability of the mice. Three days after injection with CFA, mice showed significantly decreased PWT and PWL (Fig. 5C and D), confirming the successful induction of a pain model. We then used i.t. injection to test the effects of SLR and its components on these mice. For comparison, ibuprofen (30 mg/kg), a standard NSAID, was administered *via* intraperitoneal injection as a positive control. The results demonstrated that SLR effectively mitigated pain perception, with its peak effect at 4 h post-injection. This analgesic effect was long-lasting, maintained for up to 72 h for mechanical allodynia and 48 h for thermal allodynia. In contrast, while the SeCQDs and RA groups showed a quick analgesic effect within 2 h, it only lasted for 4 h. This short duration may be due to their small size, which led to rapid distribution and metabolism. Notably, although ibuprofen provided potent and immediate relief, its efficacy lasted only 8 h. These results validated the long-lasting analgesic efficacy of SLR nanoparticles on inflammatory pain, which was attributed to the sustained-release effect of the liposomes and the synergistic action of SeCQDs and RA.

In addition to i.t. injection, i.v. injection and intraplantar (i.pl.) injection were also utilized to investigate the pain-relieving effect of SLR nanoparticles. The results showed that with both i.v. and i.pl. injections, the nanoparticles' strongest analgesic effect occurred at 4 h post-injection. However, the duration of the effect varied significantly, lasting 12 h with i.v. injection but only 4 h with i.pl. Injection (Fig. S22). It is important to note that although the concentration of the injected nanoparticles was the same for all groups, the total dose varied because of the different injection volumes (150 μL for i.v., 30 μL for i.pl., and 10 μL for i.t.). Thus, despite having the largest amount, the i.v. injection had only a moderate therapeutic effect. This may be due to the low accumulation of nanoparticles in the CNS, possibly caused by the blood-brain barrier and rapid clearance by the reticuloendothelial system organs. This aligned well with our biodistribution data for SLR after i.v. injection. In addition, the poor effect of the intraplantar injection might be because the dose of SLR nanoparticles was insufficient to completely treat the foot inflammation and inhibit the pain pathway. All results indicated that SLR nanoparticles had a pain-relieving effect on a CFA-induced inflammatory pain mouse model, but the therapeutic effect was highly dependent on the injection method, with i.t. injection having the best outcome.

### 3.9 Mechanism of SLR in alleviating inflammatory pain

**Fig. 6:**
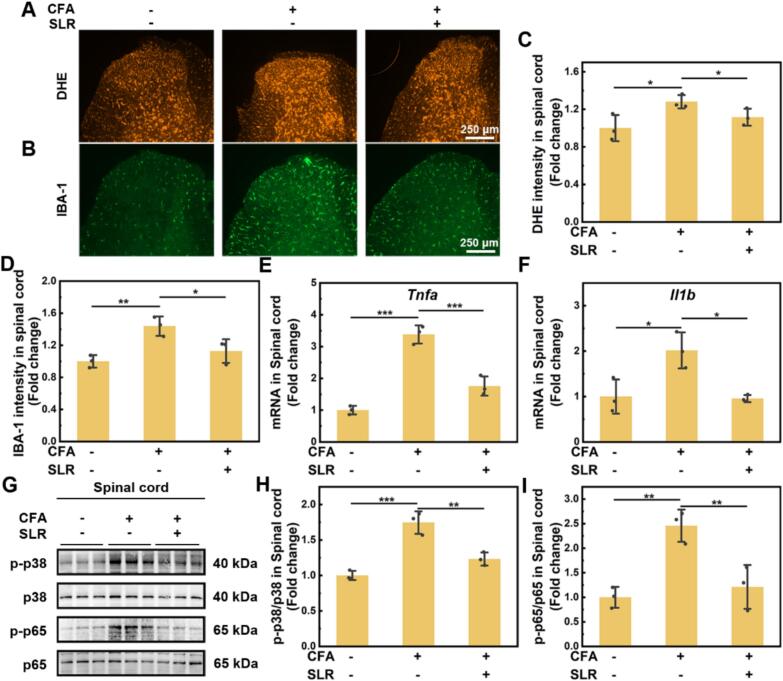
SLR nanoparticles alleviate inflammatory pain by suppressing spinal cord neuroinflammation, oxidative stress, and MAPK/NF-κB signaling. Representative images of DHE fluorescence (A) and IBA-1 (B) in the spinal dorsal horn across treatment groups (*n* = 3). Quantification of fluorescence intensity of DHE (C) and IBA-1 (D). RT-qPCR analysis of the effects on TNF-α (E) and IL-1β (F) mRNA expression in the spinal cord of mice in different treatment groups. (G) Western blot analysis of p38 and p65 phosphorylation levels in spinal cord of mice across different treatment groups. Quantitative analysis of the p-p38/p38 (H) and p-p65/p65 (I) protein expression ratios from panel G. All data are means ± SD; *n* = 3. Statistical significance (*P < 0.05, **P < 0.01, ***P < 0.001) was determined by one-way ANOVA followed by Bonferroni's *post hoc* test.

After confirming the pain relief effect of SLR, we further tried to explore the mechanism behind. Firstly, we used dihydroethidium (DHE) staining to detect the level of oxidative stress in the L4-L6 segment of the mouse spinal cord. Fluorescence microscope results (Fig. 6A and C) indicated that CFA-treated mice showed a significant increase in oxidative stress levels, while i.t. SLR injection notably reduced it. This confirms our cellular findings, demonstrating that SLR effectively eliminates RNOS in animals. The activation of microglia, the spinal cord's primary inflammatory cells, serves as a key indicator of its inflammatory state. We therefore further measured the expression levels of IBA-1, a microglia marker, in the spinal cord segment using immunofluorescence staining. The results showed that CFA injection induced the activation of microglia in spinal cord, which aligns with the understanding that inflammatory pain drives glial cell proliferation and subsequent neuroinflammation (Fig. 6B and D). The treatment of SLR greatly reduced the number of IBA-1 positive cells, indicating a reversion of activated microglia to a less inflammatory state. Subsequently, the mRNA expression levels of pro-inflammatory factors TNF-α and IL-1β in spinal cord were quantified by RT-qPCR. Fig. 6E and F showed that SLR treatment decreased the elevated levels of *Tnfa* and *Il1b* caused by CFA administration.

Based on this, we further examined the status of MAPK/NF-κB pathways in the process. Western blot results (Fig. 6G-I and S23) demonstrated that the ratios of p-p65/p65 and p-p38/p38 were significantly elevated in the spinal cord of CFA-treated mice, reflecting increased pathway activity. Importantly, SLR administration substantially reduced this activation. In addition, SLR treatment also downregulated the phosphorylation of ERK, JNK, and IκBα in CFA-treated mice (Fig. S24). Furthermore, as the biodistribution experiment showed that SLR nanoparticles reached the brain after intracethal injection, we investigated the inflammatory state and related pathways in the cerebral cortex of mice, given its involvement in the pain process. The results (Fig. S25 and S26) showed that SLR nanoparticles also significantly reversed the elevated levels of *Tnfa* and *Il1b*, along with the increased phosphorylation of p65 and p38, in the cerebral cortex CFA model mice. All the above results indicated that SLR mitigated inflammatory pain by reversing the phosphorylation of MAPK/NF-κB pathways. This action, in turn, reduces oxidative stress, alleviates inflammation, and inhibits microglial activation.

### 3.10 The effect of SLR on mouse models of neuropathic pain and the biosafety of SLR

In addition to inflammatory pain, neuropathic pain represents another significant category of chronic pain. To evaluate the therapeutic potential of SLR, we employed the CINP and SNI-induced neuropathic pain models, two gold-standard experimental paradigms. Our results demonstrated that SLR exerted a potent analgesic effect in both models (Fig. S27 and S28). The observed variations in efficacy between the two models likely stem from the differing degrees to which inflammation and oxidative stress contribute to their respective pathophysiologies.

**Fig. 7:**
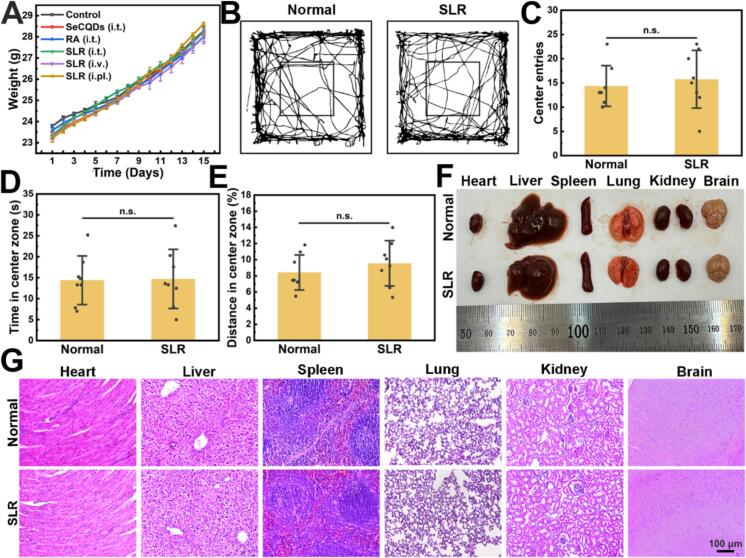
*In vivo* biosafety assessment of SLR nanoparticles. (A) Body weight growth curve of mice across different treatment groups over 15 days (All data are means ± SEM; *n* = 5). (B) Representative movement trajectories, (C) center entries, (D) time in center zoom, and (E) distance in center zoom of mice in the open field test 15 days after SLR treatment, compared to a normal control group (All data are means ± SD; *n* = 8. Statistical significance (*P < 0.05, **P < 0.01, ***P < 0.001) was determined by the Student's *t*-test). (F) Gross morphology of the heart, liver, spleen, lung, kidney, and brain of mice 15 days after i.t. administration of SLR nanoparticles, compared to a normal control group. (G) H&E staining of paraffin-embedded tissue sections from heart, liver, spleen, lung, kidney, and brain at 15 days post-administration and in the normal group.

Finally, the biosafety profile of SLR nanoparticles was evaluated. In the CFA mice model, the body weight of mice across all treatment groups remained stable, with no significant deviations from the control group (Fig. 7A). To assess potential neurotoxicity and cognitive impact, the open-field test (OFT) and novel object recognition (NOR) experiments were conducted 15 days post i.t. administration. During the OFT (Fig. 7B-E), SLR-treated mice exhibited total distances traveled and time spent in the center zone comparable to control mice, suggesting that SLR did not impair locomotor activity or induce anxiety-like behavior. Similarly, the NOR test (Fig. S29) demonstrated that the recognition index remained consistent across groups, indicating that SLR administration did not disrupt exploratory behavior or recognition memory. Beyond behavioral metrics, systemic and localized inflammatory markers were examined. Blood IgG levels, along with *Tnfa* and *Il1b* expression in the spinal cord and cerebral cortex, showed no significant differences compared to the control group (Fig. S30). Histological analysis of major organs further supported these findings. Macroscopic examination revealed no morphological abnormalities (Fig. 7F), while H&E staining demonstrated well-organized cellular structures with no evidence of inflammatory infiltration or pathological changes (Fig. 7G). To evaluate long-term toxicity, routine hematological and serum biochemical indicators were analyzed 30 days post-administration (Fig. S31 and S32). No statistically significant differences were observed in these biomarkers between the test and control groups. Collectively, these results demonstrate that SLR nanoparticles possess an acceptable biosafety profile, highlighting their potential as a biocompatible analgesic agent.

## 4 Conclusion

In summary, we successfully developed a safe and long-acting analgesic nanomedicine (SLR) by co-encapsulating SeCQDs and RA within a liposomal framework. The unique co-encapsulation leveraged the synergistic effects of SeCQDs and RA, allowing SLR nanoparticles to effectively scavenge RNOS and inhibit pro-inflammatory mediators. Through modulation of the MAPK/NF-κB signaling pathways, SLR nanomedicine regulated the pro-inflammatory microenvironment and maintained microglial quiescence. Critically, the sustained-release capabilities of the liposome led to a remarkable analgesic effect lasting up to 72 h in a CFA-induced inflammatory pain model. Our findings confirmed that this prolonged pain relief was mediated by the accumulation of SLR nanoparticles in the spinal cord and brain after i.t. administration, where they neutralize oxidative stress and regulate inflammatory signals. In addition, SLR also induced analgesic effect on CIPN and SNI-induced neuropathic pain mouse model. These results highlighted the therapeutic potential of SLR nanoparticles as a safe, sustained-release analgesic candidate for clinical translation in treating chronic pain syndromes.

## Funding

This work was supported by the 10.13039/501100001809National Natural Science Foundation of China (Grant No. 32401257 and No. 22207059) and Large Instruments Open Foundation of Nantong University (Grant No. KFJN2407).

## CRediT authorship contribution statement

**Chengfeng Zhang:** Writing – review & editing, Writing – original draft, Methodology, Investigation. **Zihan Xue:** Writing – review & editing, Writing – original draft, Methodology, Investigation. **Jingyi Wang:** Methodology, Investigation. **Jianing Li:** Methodology, Investigation. **Wanlong Qian:** Methodology, Investigation. **Xueting Wang:** Methodology. **Yong-Jing Gao:** Writing – review & editing, Methodology. **Zhongping Chen:** Writing – review & editing, Supervision, Methodology. **Faming Wang:** Writing – review & editing, Writing – original draft, Supervision, Project administration, Methodology. **Yan Zhang:** Writing – review & editing, Writing – original draft, Supervision, Project administration, Methodology, Investigation.

## Declaration of competing interest

The author(s) declare(s) that there is no conflict of interest regarding the publication of this article.

## Data Availability

Data will be made available on request.

## Appendix A Supplementary data

Supplementary material Image 1
