# Supplementary material for: A synergistic nanozyme platform breaking the neuroinflammatory-oxidative stress cycle for extended pain relief
Source: Int J Pharm X. 2026 May 5;11:100561. doi: 10.1016/j.ijpx.2026.100561 (PMC13185767; doi:10.1016/j.ijpx.2026.100561)
Supplement: Supplementary file 1 — Supplementary material [file mmc1.docx]

**Supplementary Information**

**A Synergistic Nanozyme Platform Breaking the Neuroinflammatory-Oxidative Stress Cycle for Extended Pain Relief**

Chengfeng Zhang^#^, Zihan Xue^#^, Jingyi Wang, Jianing Li, Wanlong Qian, Xueting Wang, Yong-Jing Gao, Zhongping Chen^*^, Faming Wang^*^, Yan Zhang^*^

Institute of Special Environmental Medicine, School of Public Health, Nantong University, Nantong, 226019, China

^#^The authors contributed equally to this study.

*Address correspondence to: zhangyan91@ntu.edu.cn; wangfaming1990@ntu.edu.cn; chenzp@ntu.edu.cn


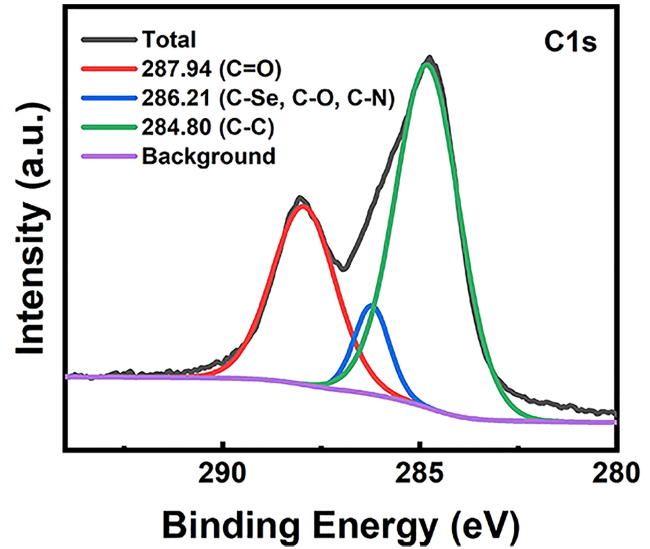


**Fig. S1**. High-resolution XPS spectrum of the C 1s peak for SeCQDs.


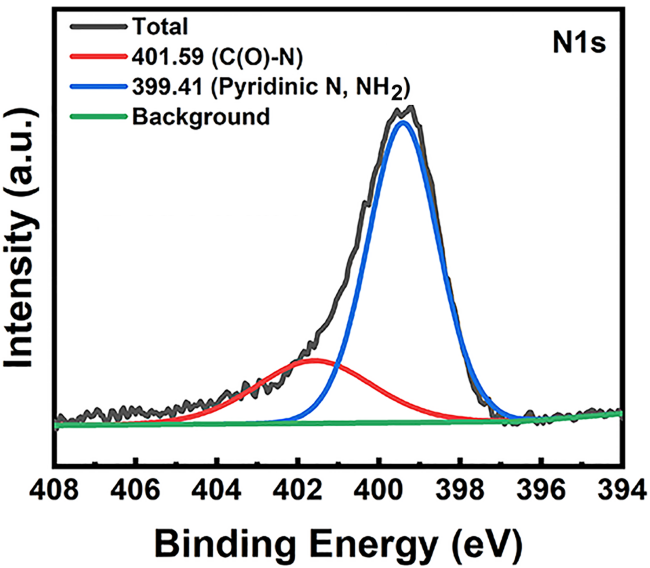


**Fig. S2**. High-resolution XPS spectrum of the N 1s peak for SeCQDs.


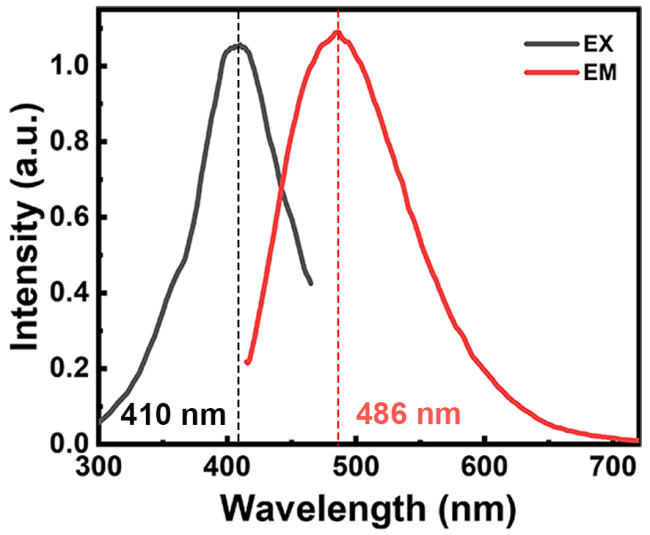


**Fig. S3**. Excitation spectrum and emission spectrum of SeCQDs.


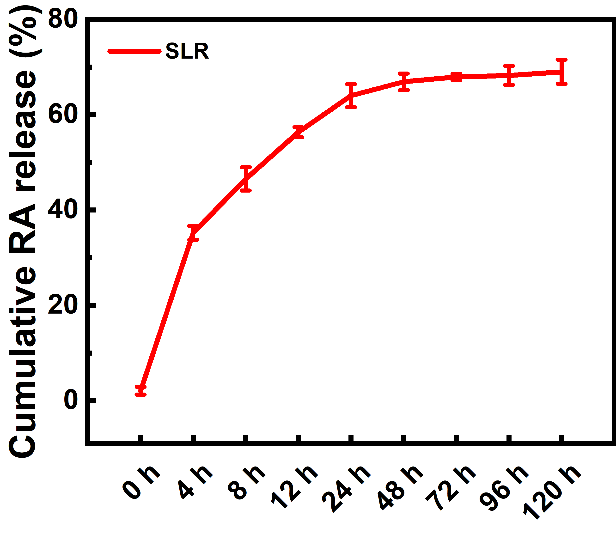


**Fig. S4**. The in vitro release characteristics of SLR were evaluated using the cumulative release of RA as the detection index.


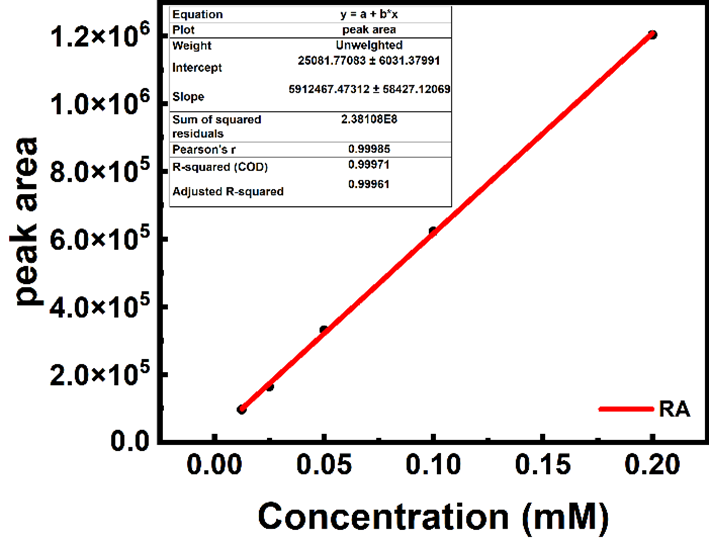


**Fig. S5**. Standard curve of the peak area of RA versus concentration, detected by HPLC in an acetonitrile-water mobile phase (65:35, containing 0.05% glacial acetic acid).

As calculated from the result of HPLC, the standard curve of RA was

Y (Peak Area) = 5912467 X (mM) + 25081 (R² = 0.9997) Formula 1.

Encapsulation efficiency (EE) % = Drug Loading Capacity of RA / Total Amount of RA×100 % Formula 2

To calculate the EE% of RA, 100 μL of SLR was mixed with 900 μL of methanol for ultrasonic membrane disruption. After filtration through a 200 nm filter membrane, HPLC analysis was performed, and the peak area was approximately 306,875. Substitute the measured peak area into Formula 1 and Formula 2, and the calculated encapsulation efficiency of RA was 22.2%.


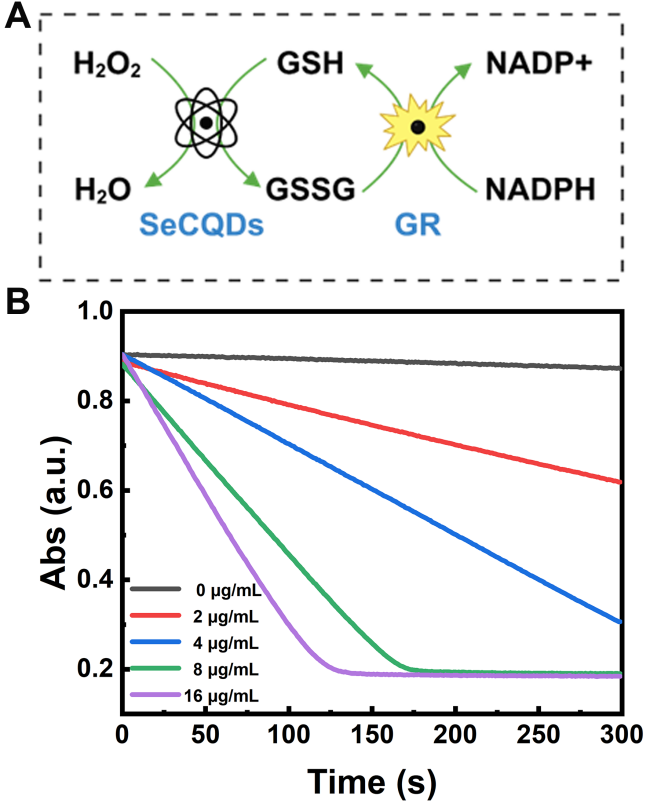


**Fig. S6**. GPx-like enzyme activity of SeCQDs. (A) Schematic diagram of the detection mechanism for GPx-like enzyme activity of SeCQDs. (B) Time-dependent absorbance changes of NADPH with different concentrations of SeCQDs.


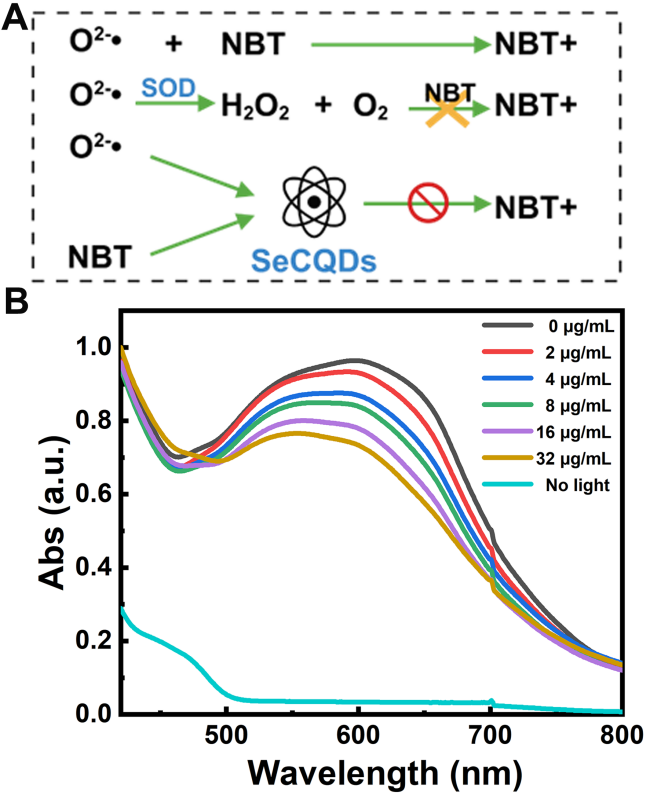


**Fig. S7**. SOD-like enzyme activity of SeCQDs. (A) Schematic diagram of the detection mechanism for SOD-like enzyme activity of SeCQDs. (B) Scavenging efficiency of superoxide radicals with different concentrations of SeCQDs.


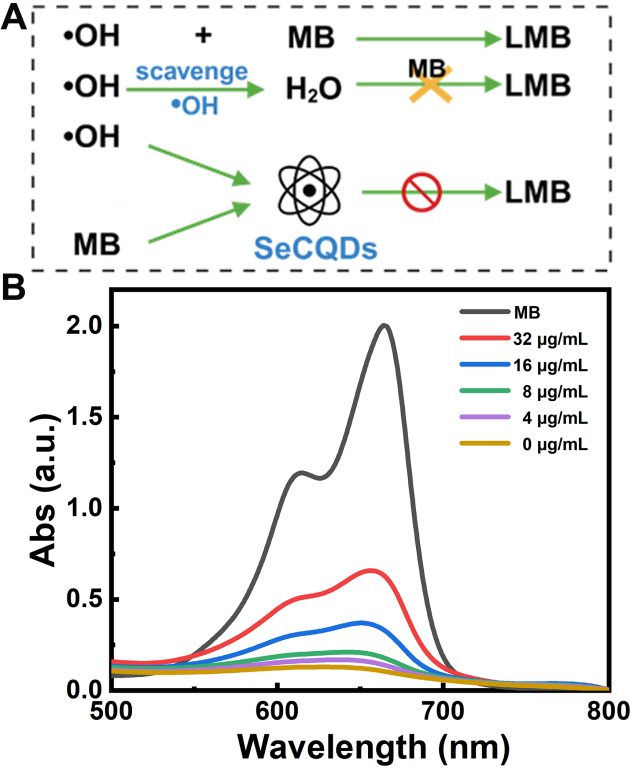


**Fig. S8**. Hydroxyl radical scavenging activity of SeCQDs. (A) Schematic diagram of the detection mechanism for hydroxyl radical scavenging activity of SeCQDs. (B) Scavenging efficiency of hydroxyl radical with different concentrations of SeCQDs.


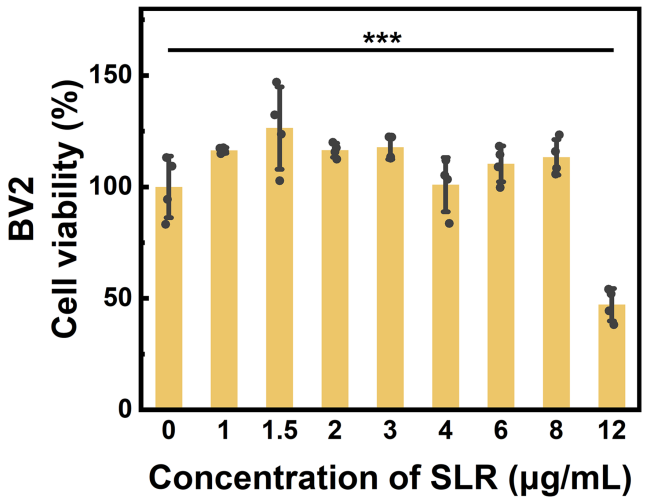


**Fig. S9**. The cell viability of BV2 cells after incubating them with different concentrations of SLR nanoparticles for 24 h. All data are means ± SD; n = 4. Statistical significance (*P < 0.05, **P < 0.01, ***P < 0.001) was determined by one-way ANOVA followed by Bonferroni's post hoc test.


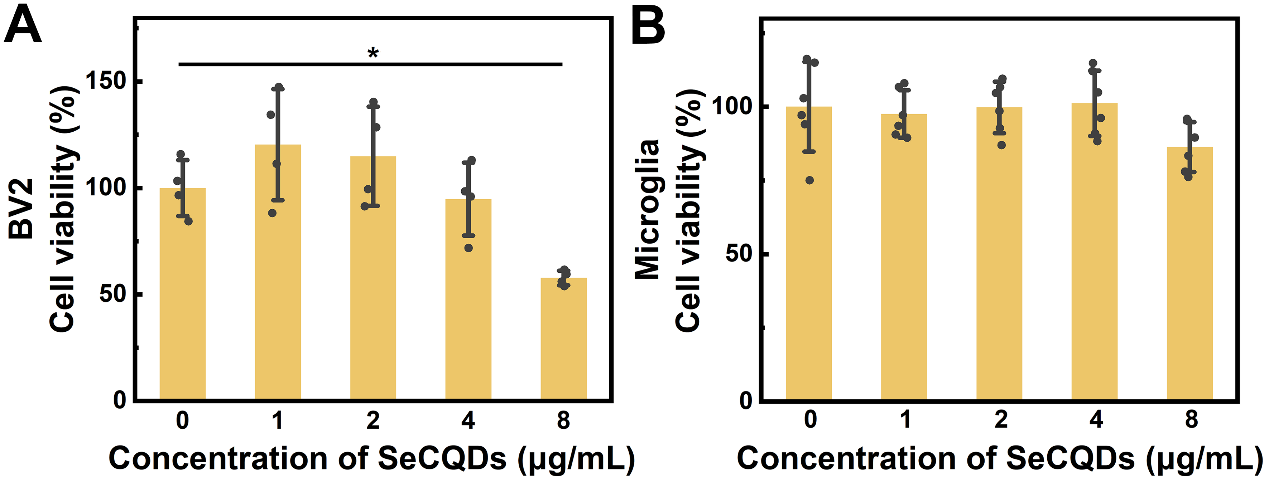


**Fig. S10**. The cell viability of BV2 cells (A) (n=4) and primary microglia cells (B) (n=6) after incubating them with different concentrations of SeCQDs for 24 h. All data are means ± SD. Statistical significance (*P < 0.05, **P < 0.01, ***P < 0.001) was determined by one-way ANOVA followed by Bonferroni's post hoc test.


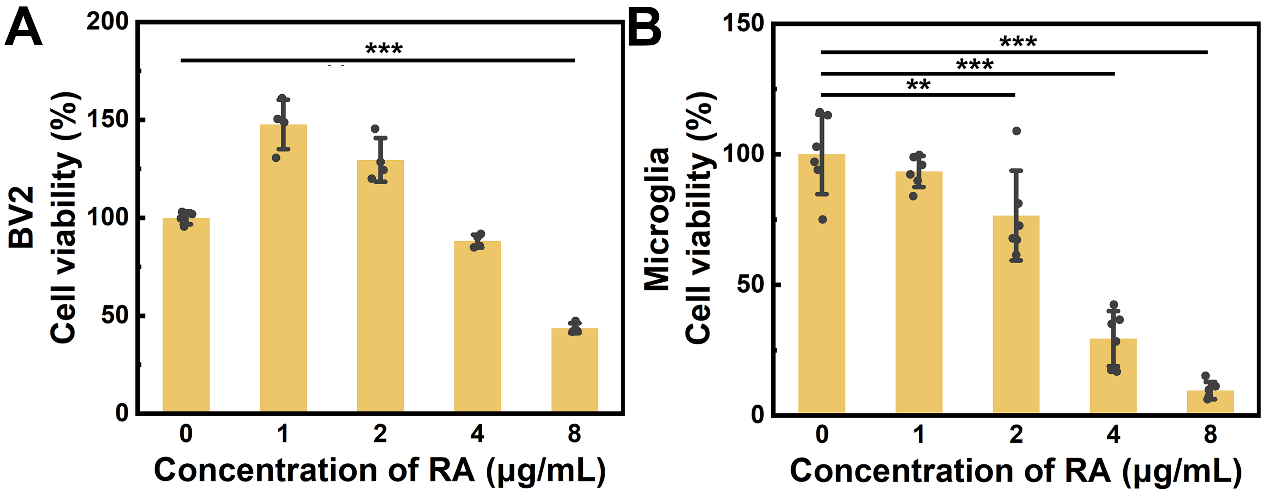


**Fig. S11**. The cell viability of BV2 cells (A) (n=4) and primary microglia cells (B) (n=6) after incubating them with different concentrations of RA for 24 h. All data are means ± SD. Statistical significance (*P < 0.05, **P < 0.01, ***P < 0.001) was determined by one-way ANOVA followed by Bonferroni's post hoc test.


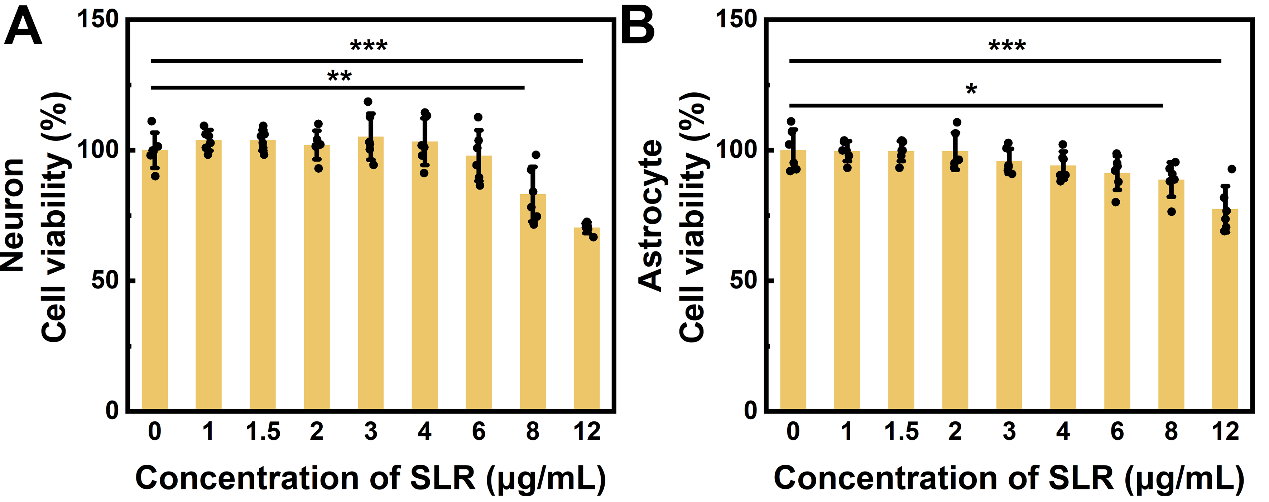


**Fig. S12**. The cell viability of neurons (A) (n = 6) and primary astrocytes (B) (n = 6) after incubating them with different concentrations of SLR for 24 h. All data are means ± SD. Statistical significance (*P < 0.05, **P < 0.01, ***P < 0.001) was determined by one-way ANOVA followed by Bonferroni's post hoc test.


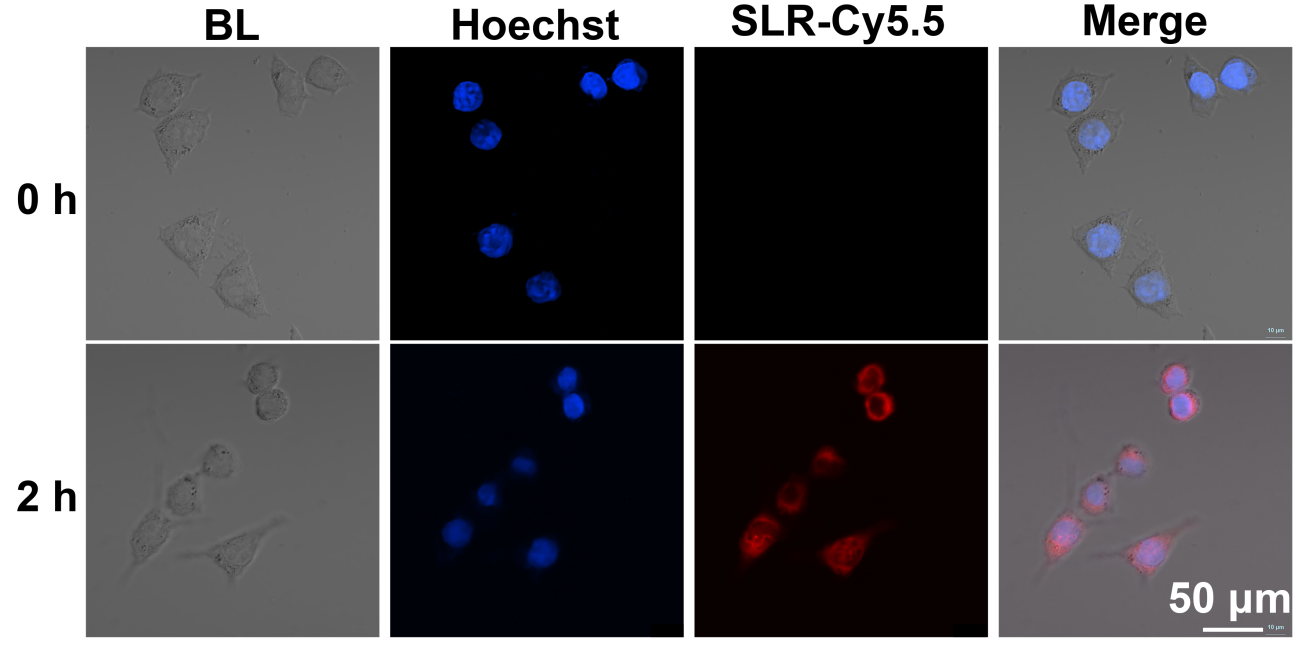


**Fig. S13**. Fluorescence microscopy images of BV2 cell incubated with SLR-Cy5.5 nanoparticles.


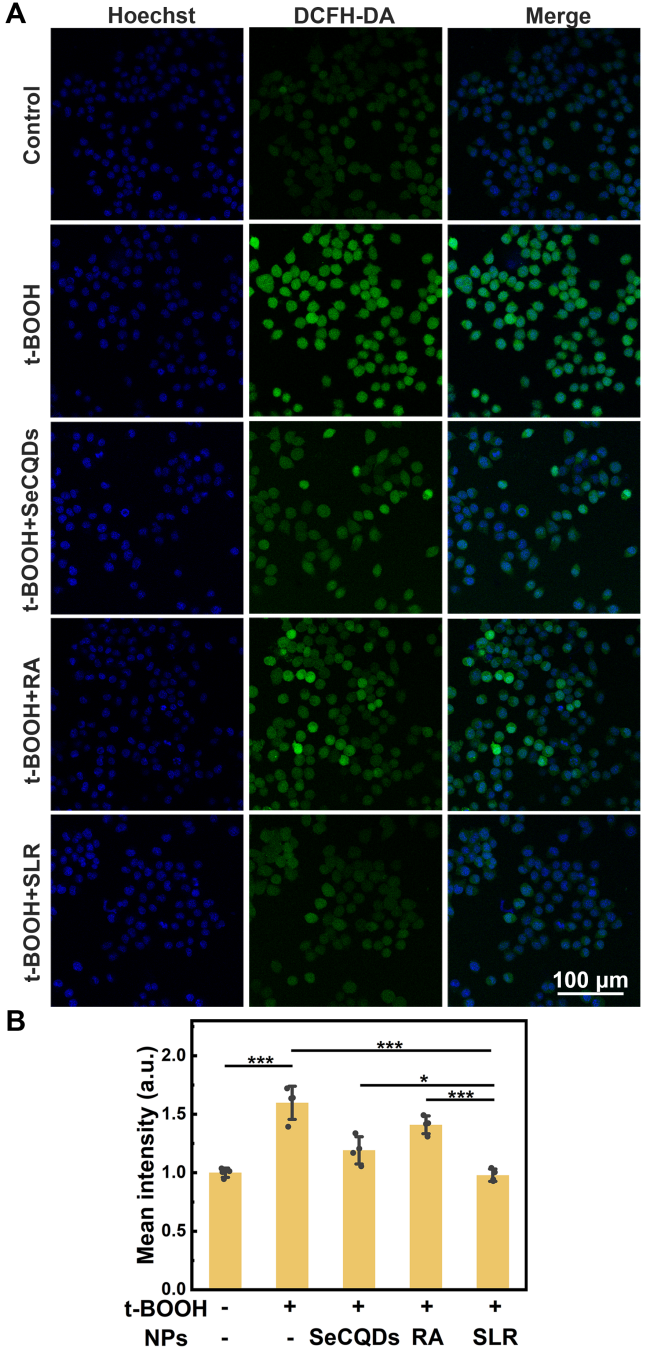


**Fig. S14**. (A) Confocal microscopy images illustrating the reduction of RNOS in t-BOOH-stimulated BV2 cells following various treatments, as detected by the DCFH-DA probe. (B) Quantitative analysis of the mean DCFH-DA fluorescence intensity corresponding to RNOS levels. All data are means ± SD; n = 4. Statistical significance (*P < 0.05, **P < 0.01, ***P < 0.001) was determined by one-way ANOVA followed by Bonferroni's post hoc test.


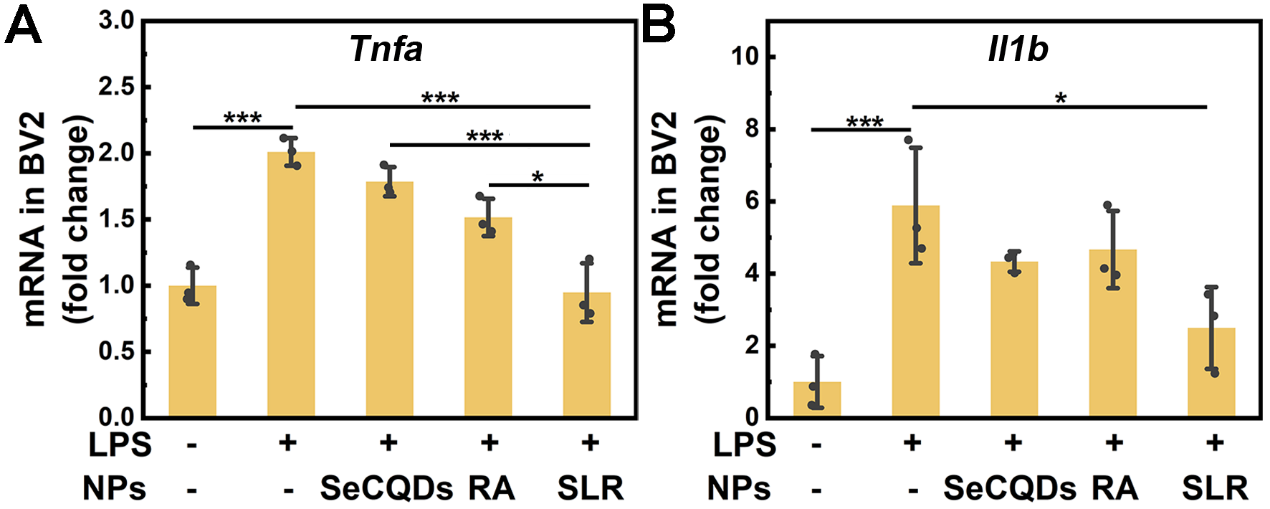


**Fig. S15**. RT-qPCR analysis of nanoparticle effects on TNF-α (A) and IL-1β (B) mRNA expression in LPS-stimulated BV2 cells. All data are means ± SD; n = 3. Statistical significance (*P < 0.05, **P < 0.01, ***P < 0.001) was determined by one-way ANOVA followed by Bonferroni's post hoc test.


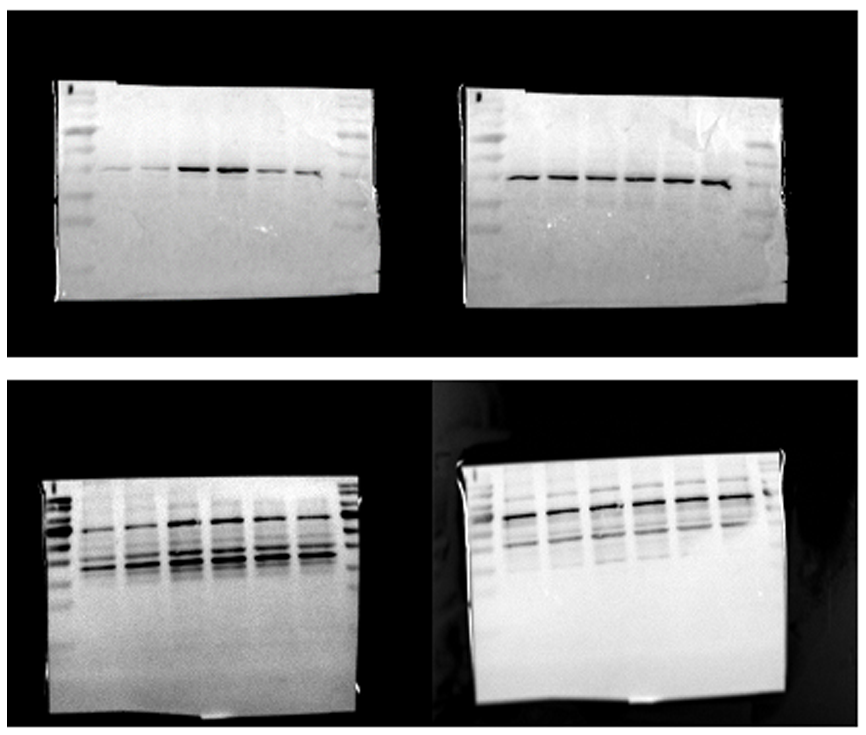


**Fig. S16.** Full membrane blot corresponding to the Western blot shown in Fig. 4A.

**
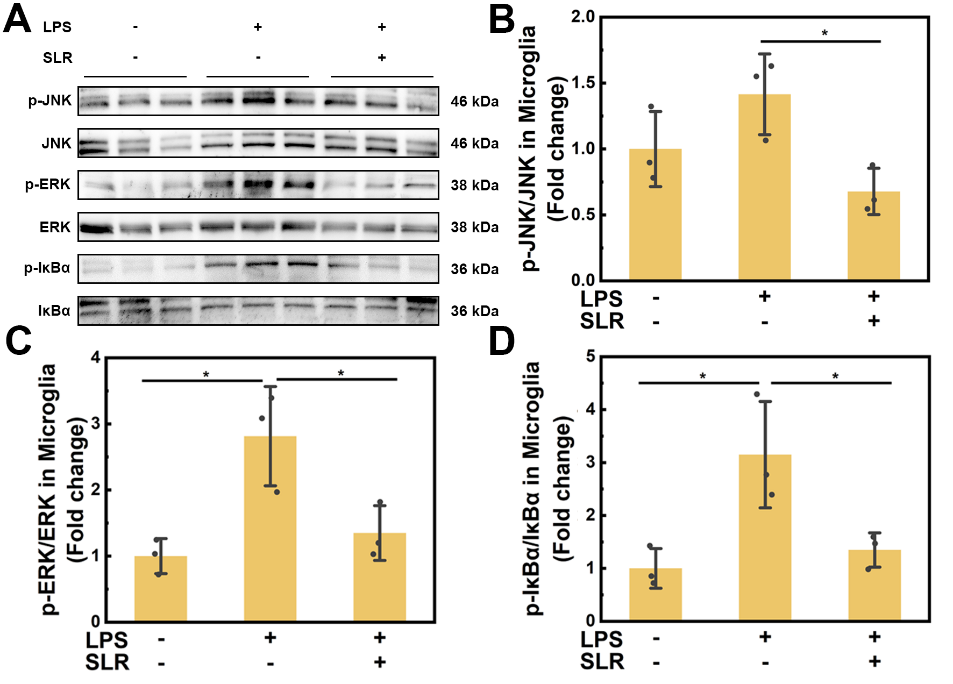
**

**Fig. S17.** Phosphorylation of JNK, ERK, and IκBα in the microglia. (A) Western blot analysis of p-JNK, JNK, p-ERK, ERK, p-IκBα and IκBα levels across experimental treatment groups. Quantitative analysis of the p-JNK/JNK (B), p-ERK/ERK (C), p-IκBα/IκBα (D) protein expression ratios from panel A. All data are means ± SD; n = 3. Statistical significance (*P < 0.05, **P < 0.01, ***P < 0.001) was determined by one-way ANOVA followed by Bonferroni’s post hoc test.


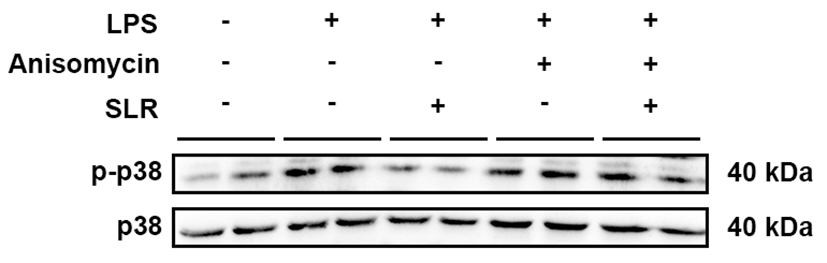


**Fig. S18.** Phosphorylation of p38 in the microglia after different treatments.


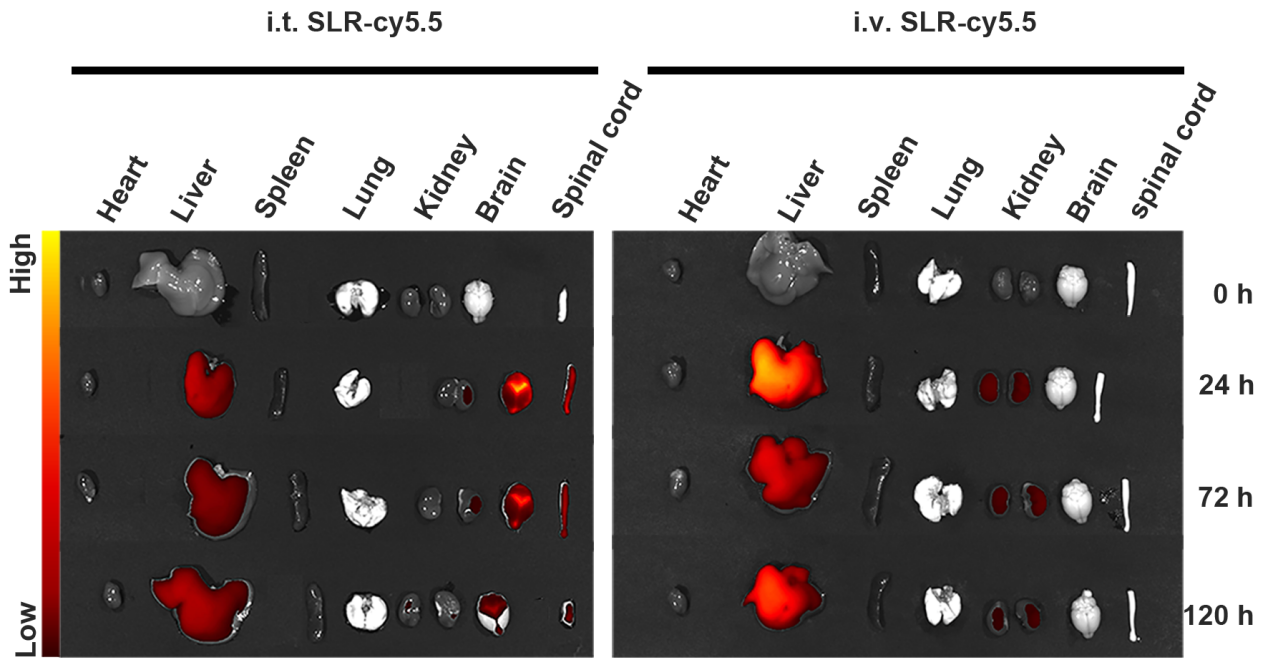


**Fig. S19**. Fluorescence imaging of the heart, liver, spleen, lung, kidney, brain, and spinal cord at various time points following i.t. or i.v. injection of SLR-Cy5.5 nanoparticles in mice.


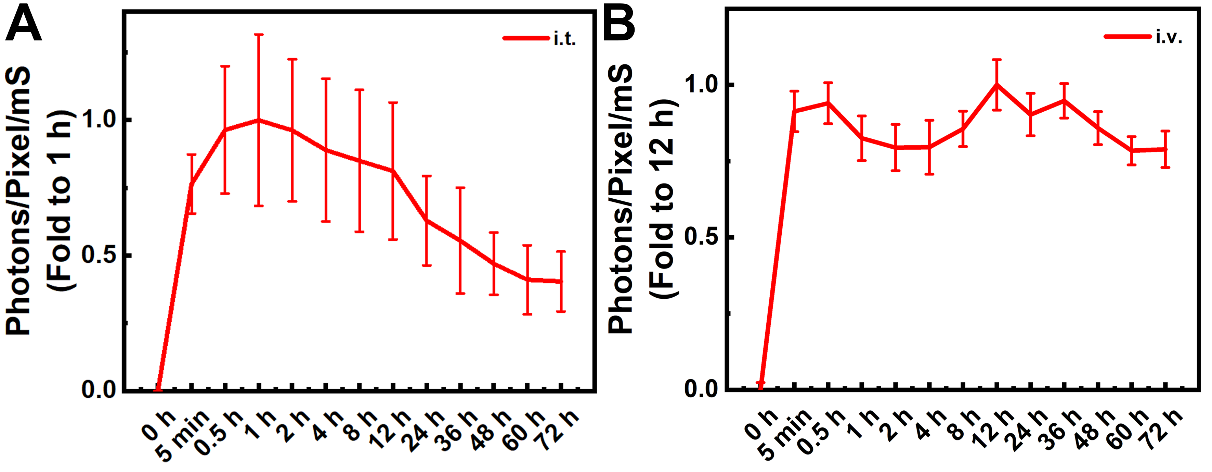


**Fig. S20.** Distribution and clearance kinetics of SLR-Cy5.5. Quantification of radiant efficiency derived from *in vivo* fluorescence imaging. Fluorescent signal of mice after intrathecal (i.t.) injection (A) and intravenous (i.v.) injection (B). n = 3.


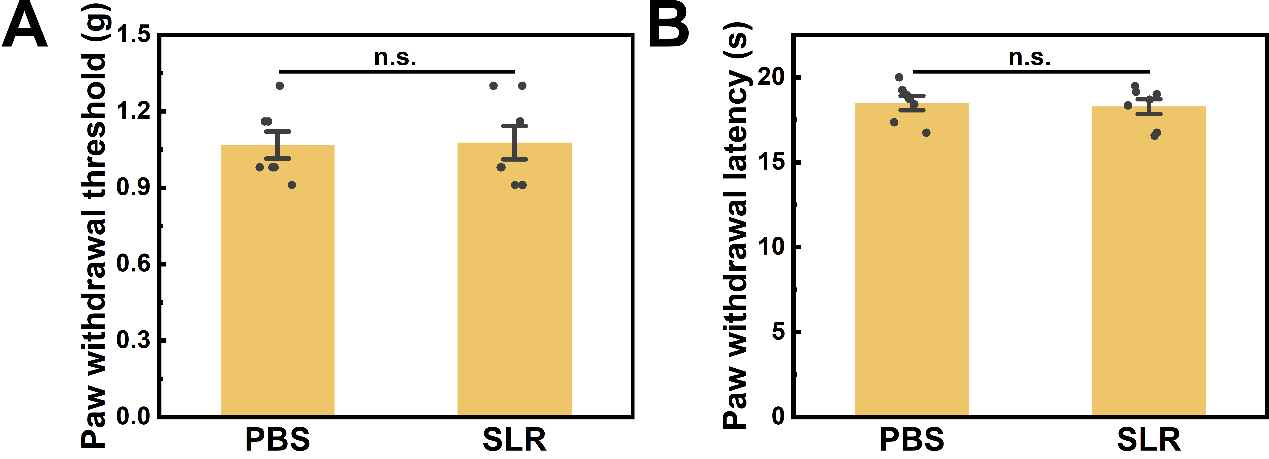


**Fig. S21**. The effects of intrathecally administered SLR nanoparticles (1 day post-injection) on (A) mechanical allodynia and (B) thermal hyperalgesia in mice, compared to a normal control group. All data are means ± SEM; n = 7. Statistical significance (*P < 0.05, **P < 0.01, ***P < 0.001) was determined by the Student’s t-test.


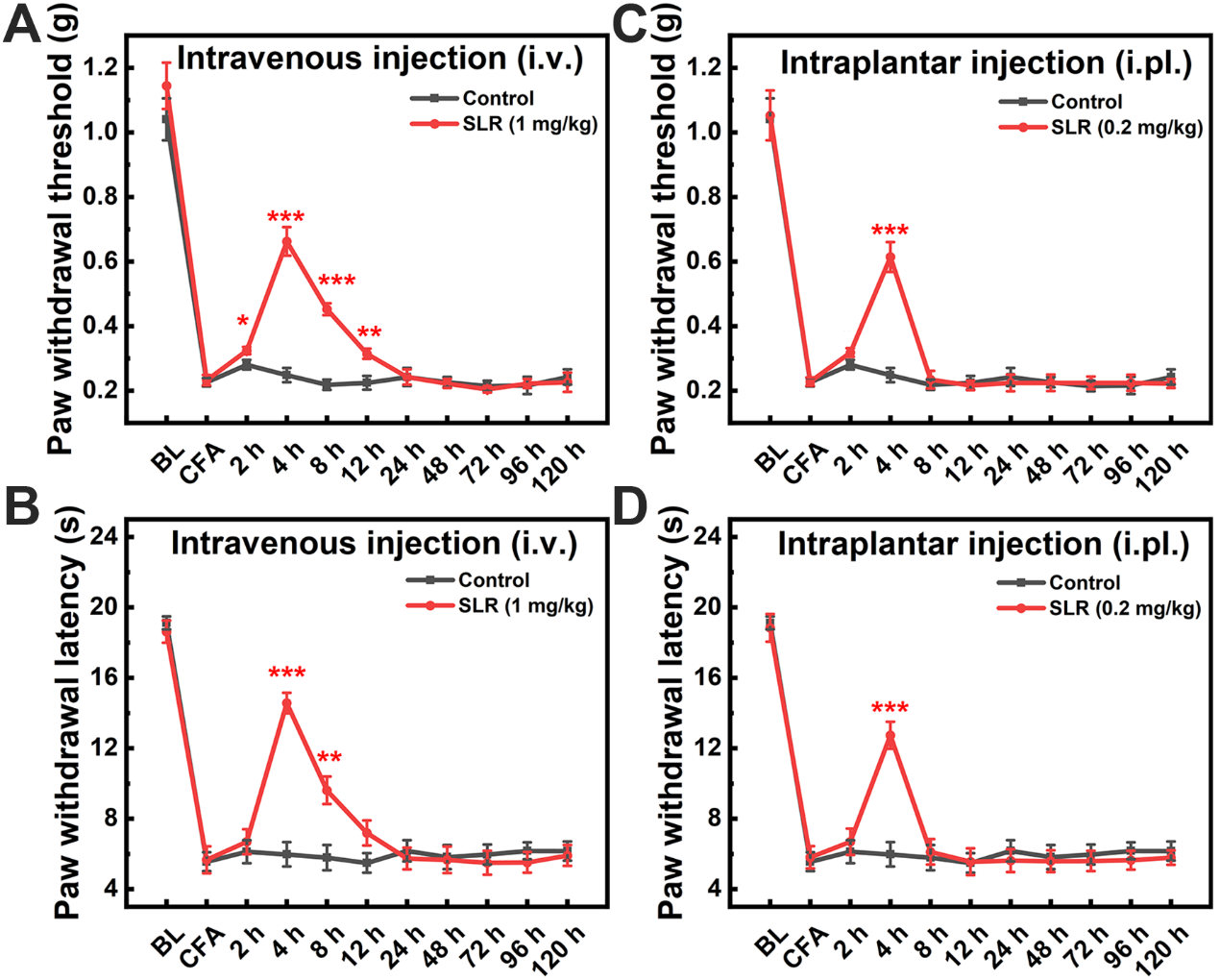


**Fig. S22**. Effect of SLR nanoparticles on CFA-induced inflammatory pain. Effects of intravanous injection of SLR nanoparticles on mechanical allodynia (A) and thermal hyperalgesia (B) in CFA-induced mouse. Effects of intraplantar injection of SLR nanoparticles on mechanical allodynia (C) and thermal hyperalgesia (D) in CFA-induced mouse (n=5). All data are means ± SEM; n = 5. Statistical significance (*P < 0.05, **P < 0.01, ***P < 0.001) was determined by two-way mixed ANOVA followed by Tukey’s post-hoc test.


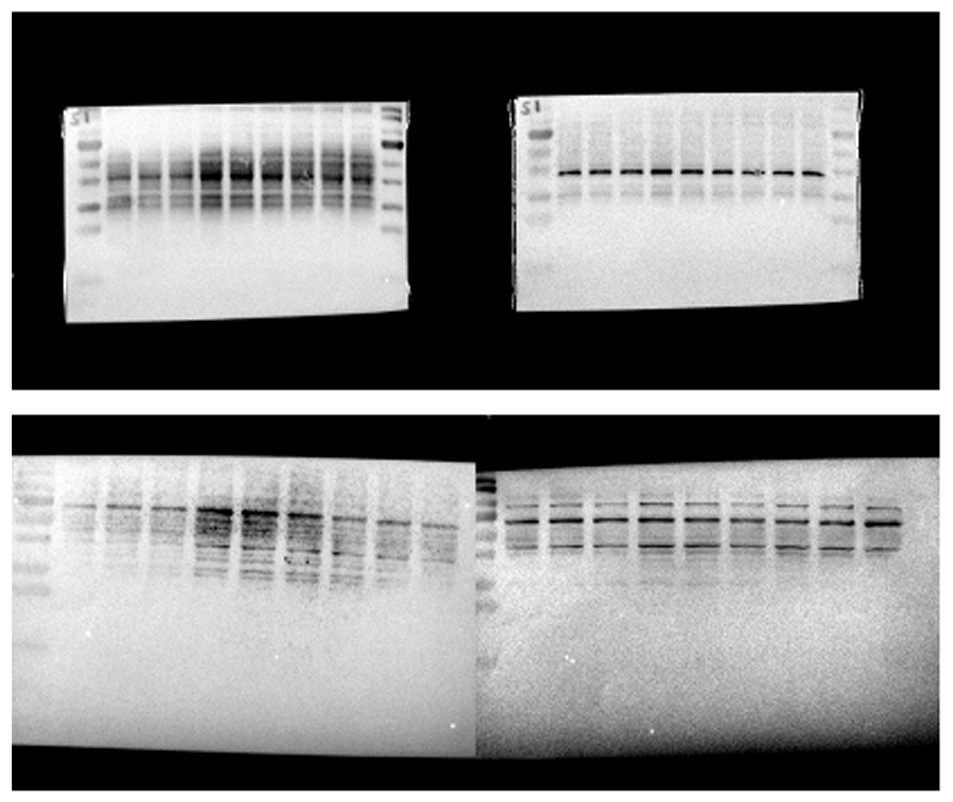


**Fig. S23.** Full membrane blot corresponding to the Western blot shown in Fig. 6G.


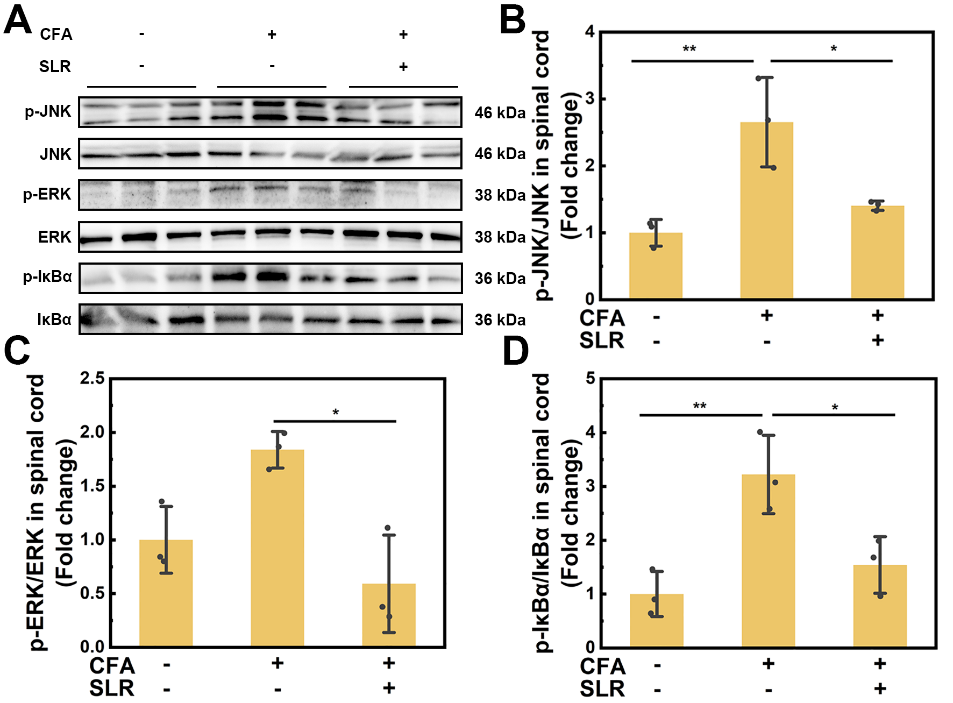


**Fig. S24.** Phosphorylation of JNK, ERK, and IκBα in the mouse spinal cord. (A) Western blot analysis of p-JNK, JNK, p-ERK, ERK, p-IκBα and IκBα levels across experimental treatment groups. Quantitative analysis of the p-JNK/JNK (B), p-ERK/ERK (C), p-IκBα/IκBα (D) protein expression ratios from panel A. All data are means ± SD; n = 3. Statistical significance (*P < 0.05, **P < 0.01, ***P < 0.001) was determined by one-way ANOVA followed by Bonferroni’s post hoc test.


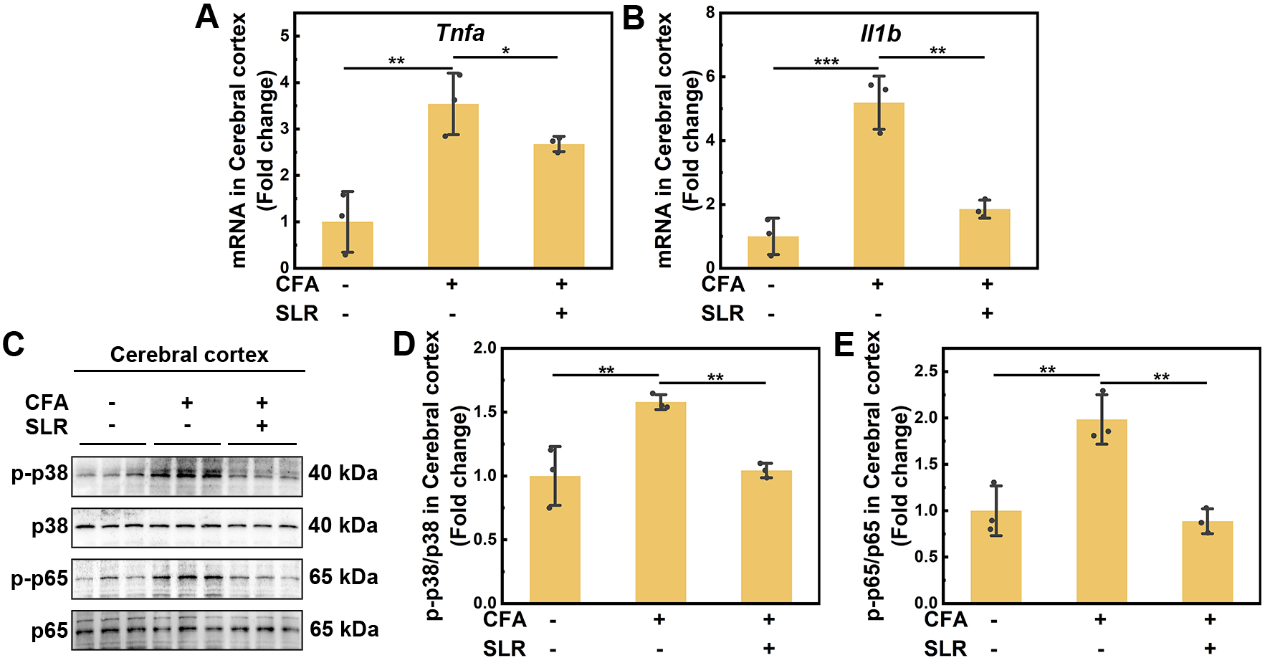


**Fig. S25**. RT-qPCR analysis of the effects on TNF-α (A) and IL-1β (B) mRNA expression in the cerebral cortex of mice in different treatment groups. (C) Western blot analysis of p38 and p65 phosphorylation levels in cerebral cortex of mice across different treatment groups. Quantitative analysis of the p-p38/p38 (D) and p-p65/p65 (E) protein expression ratios from panel C. All data are means ± SD; n = 3. Statistical significance (*P < 0.05, **P < 0.01, ***P < 0.001) was determined by one-way ANOVA followed by Bonferroni's post hoc test.


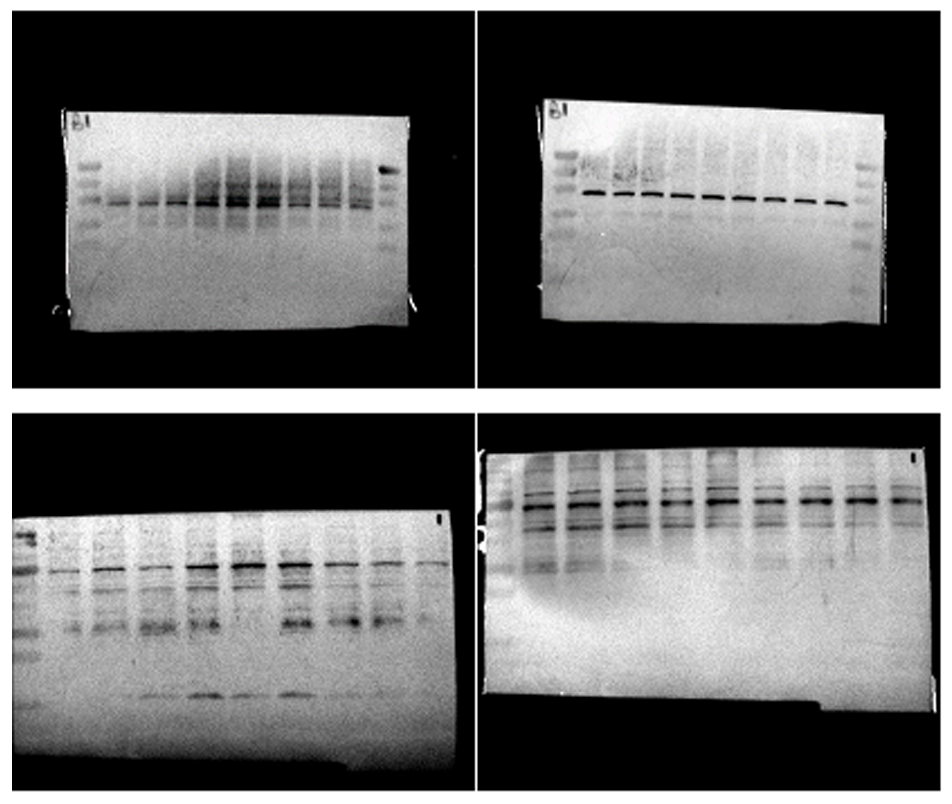


**Fig. S26.** Full membrane blot corresponding to the Western blot shown in Fig. S25C.


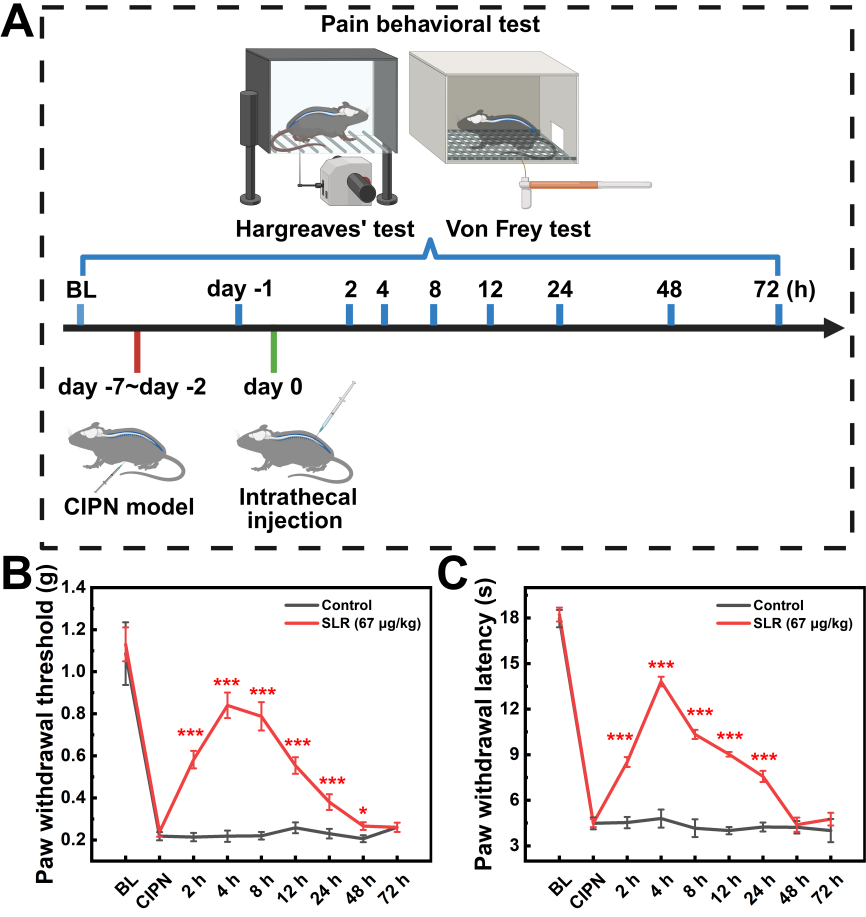


**Fig. S27.** (A) Schematic illustration of the CIPN pain model and behavioral testing. Analgesic effects of i.t. administration of SLR on mechanical allodynia (B) and thermal hyperalgesia (C) in CIPN-mice. (n=5). Statistical significance (*P < 0.05, **P < 0.01, ***P < 0.001) was determined by two-way mixed ANOVA followed by Tukey’s post-hoc test.


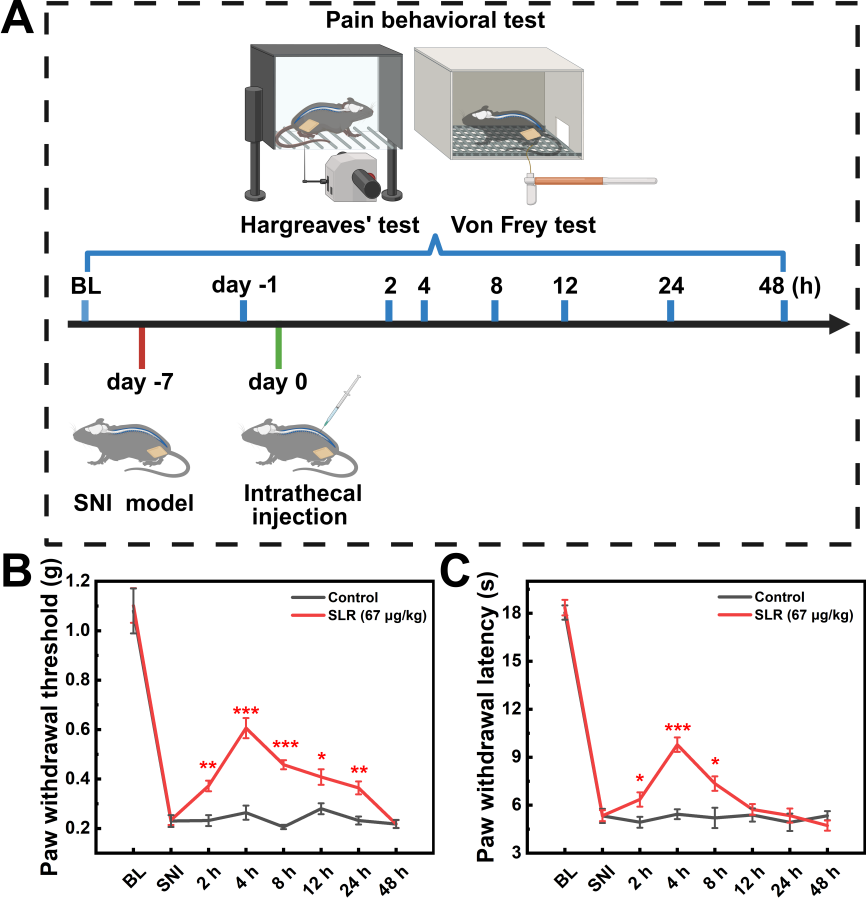


**Fig. S28**. (A) Schematic illustration of the SNI-induced neuropathic pain model and behavioral testing. Analgesic effects of i.t. administration of SLR on mechanical allodynia (B) and thermal hyperalgesia (C) in SNI-mice. (n=5). All data are means ± SEM; Statistical significance (*P < 0.05, **P < 0.01, ***P < 0.001) was determined by two-way mixed ANOVA followed by Tukey’s post-hoc test.


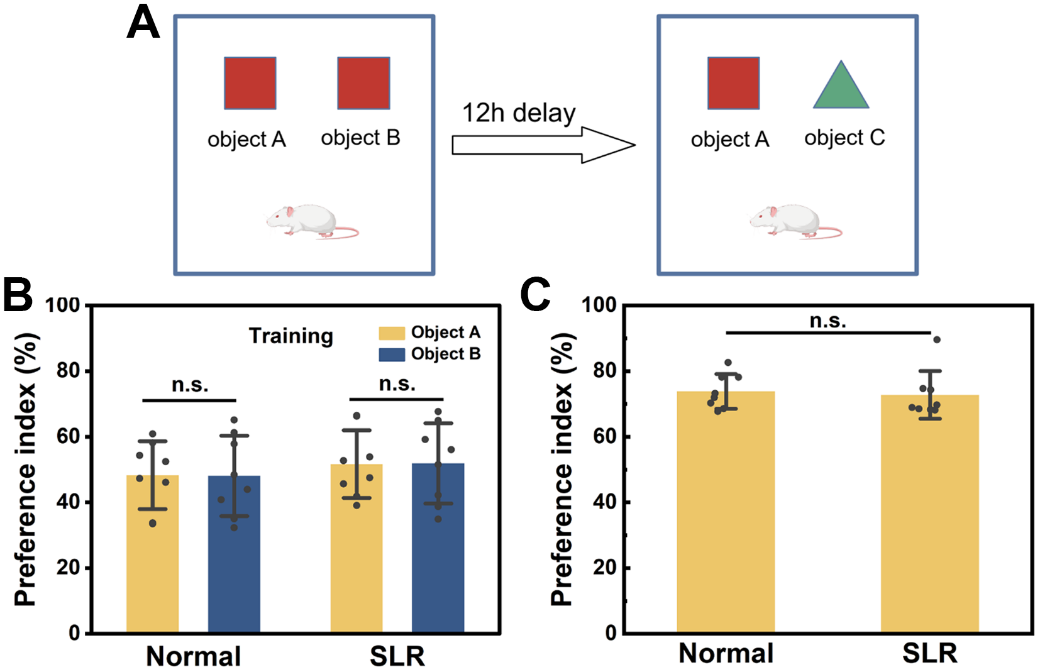


**Fig. S29.** 15 days after i.t. administration of SLR nanoparticles, mice were tested with novel object recognition (NOR) experiments (A). NOR test was performed to assess preference indices during the training phase (B) and testing phase (12-h delay) (C). n = 8. All data are means ± SD; Statistical significance (*P < 0.05, **P < 0.01, ***P < 0.001) was determined by the Student’s t-test.


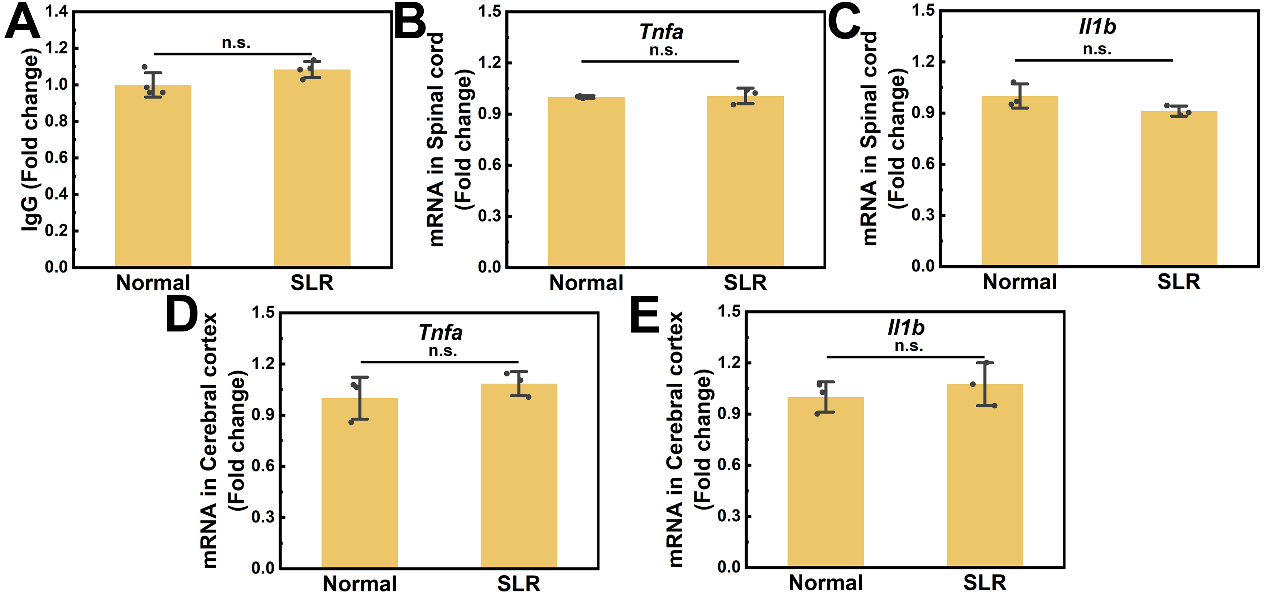


**Fig. S30.** The blood IgG level (A) (n = 4), spinal cord TNF-α (B) and IL-1β (C) mRNA expression (n = 3), cerebral cortex TNF-α (D) and IL-1β (E) mRNA expression (n = 3) in mice following i.t. administration of SLR nanoparticles (15 days post-injection) versus a normal control group. All data are means ± SD; Statistical significance (*P < 0.05, **P < 0.01, ***P < 0.001) was determined by the Student’s t-test.


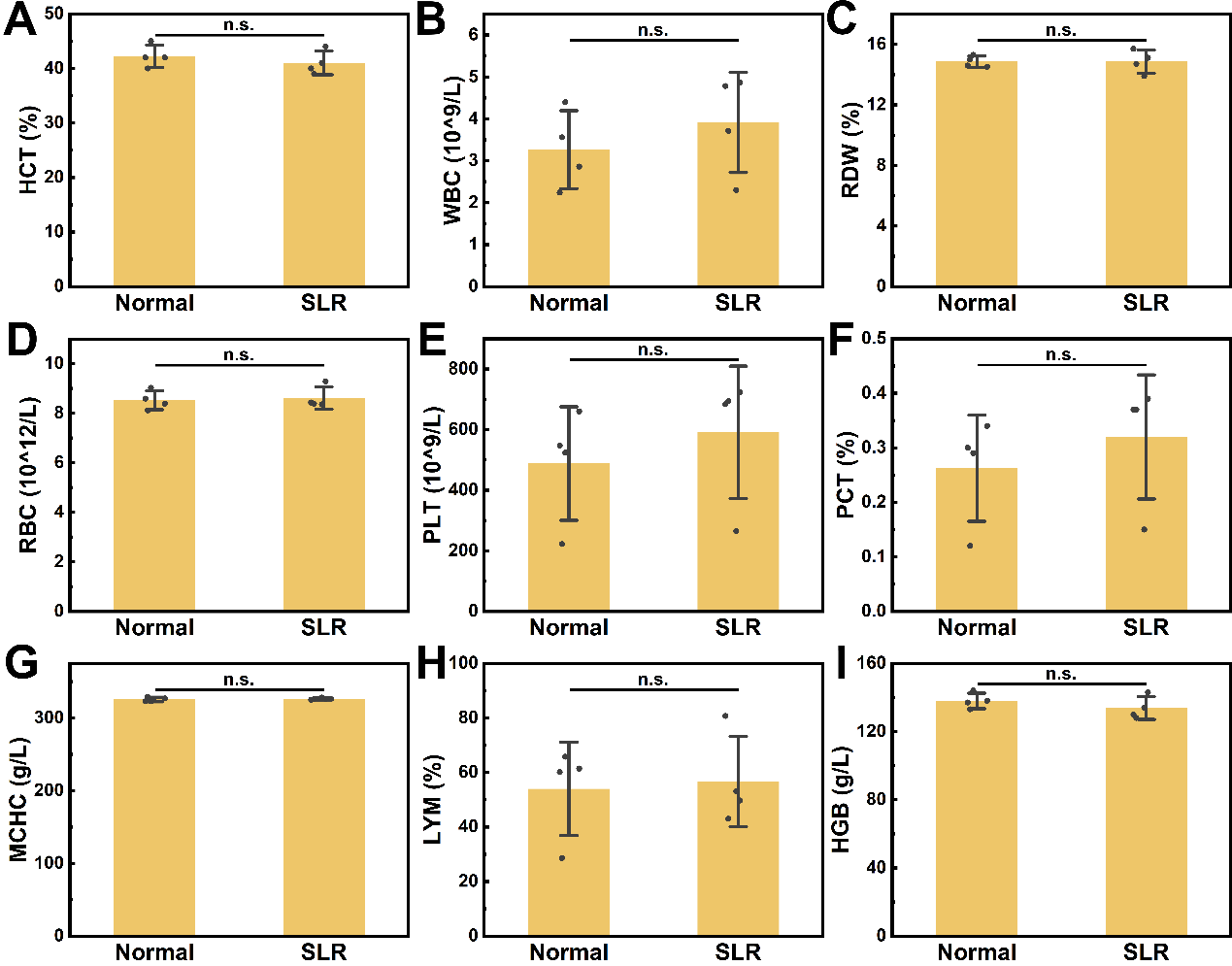


**Fig. S31**. The biosafety evaluation of SLR nanoparticles administration in mice. Comparison of blood routine parameters in mice following i.t. administration of SLR nanoparticles (30 days post-injection) versus a normal control group. All data are means ± SD; n = 4. Statistical significance (*P < 0.05, **P < 0.01, ***P < 0.001) was determined by the Student’s t-test.


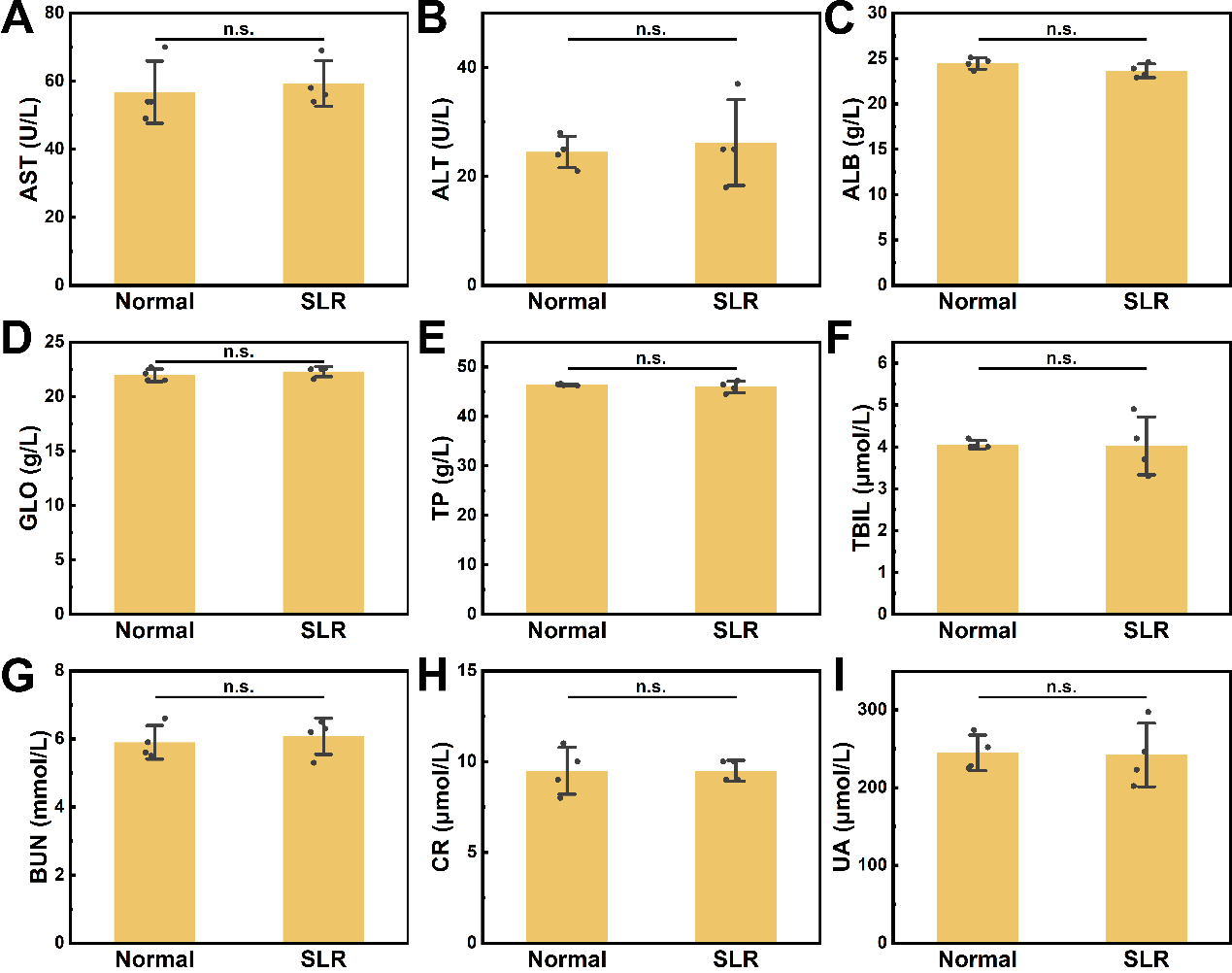


**Fig. S32**. The biosafety evaluation of SLR nanoparticles administration in mice. Comparison of serum biochemical indicators for liver function and renal function in mouse following i.t. administration of SLR nanoparticles (30 days post-injection) versus a normal control group (n=4). All data are means ± SD; n = 4. Statistical significance (*P < 0.05, **P < 0.01, ***P < 0.001) was determined by the Student’s t-test.
